# Welfare state decommodification and population health

**DOI:** 10.1371/journal.pone.0272698

**Published:** 2022-08-31

**Authors:** Olivier Jacques, Alain Noël

**Affiliations:** 1 Département de Gestion, Évaluation et Politique de Santé, École de Santé Publique, Université de Montréal, Montréal, Québec, Canada; 2 Département de Science Politique, Université de Montréal, Montréal, Québec, Canada; State Health Resource Center, Chhattisgarh, INDIA

## Abstract

A generous welfare state decommodifies social relations and frees citizens from relying excessively on markets. We argue that decommodification is associated with population health in two ways: directly, as it provides better social protection to households and indirectly, as it mitigates health-damaging labour market polarization and reduces the incidence of labour market risks. Using time-series cross-sectional quantitative analysis for 21 OECD countries from 1971 to 2010, we observe a negative relationship between decommodification and the age-standardized death rate. We then analyze three correlates of decommodification—income redistribution, labour market polarization and the reduction of labour market risk incidence—and find that only the latter two are associated with population health. Higher labour market polarization, measured by the share of market income allocated to the richest decile relative to the share of the poorest decile, is associated with a higher death rate. A new measure of risk reduction, the degree to which the welfare state reduces the prevalence of large income losses, is also associated with lower death rates, especially for men. Welfare state decommodification thus contributes to population health directly, and indirectly, via the attenuation of labour market polarization and the mitigation of labour market risks.

## Introduction

Numerous studies have analysed the association between the welfare state and population health. Previous research has established a relationship between overall welfare state generosity and population health [1–3] and connected the benefits provided by specific social programs such as social assistance or unemployment insurance to health outcomes [4–7] or to well-being [8]. However, the exact mechanisms associating the welfare state to health outcomes remain undetermined. Because the welfare state has well-established redistributive consequences, one may think that social programs improve population health because they reduce inequality [9]. However, several studies question the relationship between the overall distribution of disposable income and population health [10, 11].

We argue that welfare states and social programs free citizens from relying excessively on markets [2]. In other words, a generous welfare state decommodifies social relations [12], leaving individuals less vulnerable to market forces and better able to make healthy choices. Decommodification is the key mechanism linking the welfare state to population health, directly and indirectly. As it directly provides better social protection to households and indirectly reduces labour market polarization and the incidence of labour market risks, decommodification makes for more secure, healthier lives.

To assess this theory, we build time-series cross-sectional models for 21 OECD countries between 1971 and 2010, using as a dependent variable the age-standardized death rate for both women and men, as previous studies find significant gender differences in the determinants of health [13, 14]. Decommodification, which we conceive as an outcome of the welfare state [15], is measured through the welfare state generosity index designed by Lyle Scruggs, Detlef Jahn, and Kati Kuitto [16; see also 17]. Labour market polarization is assessed with the ratio of market income between the richest and the poorest 10% of the distribution (p90p10). We consider the reduction of risk incidence with a new measure of the degree to which taxes and transfers reduce the frequency of major losses in household incomes [18]. Building on the important studies of Lundberg et al. [1] and Beckfield and Bambra [2], we observe a positive relationship between welfare state generosity and population health. Our main contribution is to specify how decommodification relates to health outcomes.

## Material and methods

### Theory and hypotheses

Decommodification is an outcome of generous welfare states. Strong social programs provide individuals with resources and options allowing them not to accept a job at any condition [12]. The welfare state also provides public transfers and services enhancing citizens’ autonomy and security, their capacity to stay in good health, and even their subjective well-being [19]. Social programs cushion the impact of individual life events on health [20]. Job displacement, for instance, is associated with poorer health in the long-term [21], but more generous unemployment insurance and more protective labour market regulations, clear forms of decommodification, reduce the association between unemployment on health [6, 22]. Similarly, Levecque et al. [23] find that the welfare state attenuates the consequences of economic hardship for health. However, Voßemer et al. [8] observe that generous unemployment insurance improves well-being but not self-reported health.

We know from the literature on population health that welfare state regimes [24] or social expenditures are associated with better health outcomes, whether one is comparing OECD countries, US states, or Canadian provinces [25–27]. Bambra [28] establishes a cross-sectional correlation between the welfare state generosity index and infant mortality, whereas Beckfield and Bambra [2] reach similar conclusions by extending the analysis with time-series cross-sectional modelling. Studies on specific social transfers such as social assistance or unemployment insurance reinforce these findings associating benefit generosity with positive health outcomes [4–7, 29].

The original work of Esping-Andersen on decommodification was criticized by feminist scholars for neglecting gender [30]. Esping-Andersen admitted the problem and introduced the concept of familialism to take gender into account [31]. Bambra et al. [32] established that indeed gender differences in self-reported health are related to welfare state regimes. More generally, we can expect gender differences in the relationship between decommodification and health. The association of welfare state generosity and health should be stronger for women than for men, since women rely more than men on health care, social services, programs to support families, and basic pensions [13]. Dahl and van der Wel [33], for instance, find that social expenditures are associated with lower health inequalities among women, more than among men. Women also receive less from employer-based contributory pension plans, are more likely to be poor in old age, and benefit more from decommodified basic pensions [34]. Björn Högberg [14] observes that while good standard pensions benefit men’s health, generous minimum pensions improve women’s health in old age, as does higher spending on eldercare (see also [35]). We thus formulate the following hypotheses about the direct and gendered relationship between decommodification and death rates:

H_*1a*_: *Welfare state generosity is associated with lower death rates*

H_*1b*_: *And this association is stronger for women than for men*

An intuitive interpretation of this hypothesis would relate the association between the welfare state and health to income redistribution [9, 20, 21]. Indeed, a vast and largely consensual literature establishes a link between the welfare state and redistribution [36]. Several studies, however, contend that there is no association between overall redistribution and population health [10, 11, 26]. We argue that the association between welfare state generosity and health stems not so much from the total redistributive effect of the welfare state but rather from the provision of better social protection. We thus expect to see a positive association between decommodification and population health, not mediated by income redistribution.

H_1c_: *Welfare state generosity is associated with lower death rates even when controlling for income redistribution*

In addition to the direct association between decommodification and health, we expect two indirect connections. First, decommodification transforms the labour market: the institutional configuration of a welfare state regime structures the distribution and conditions of employment [12, 31]. We argue that decommodification reduces labour market polarization and lowers labour market risks such as large income losses due to unemployment, illness, or the loss of a spouse [18]. In turn, both factors are associated with better health.

Post-industrial, globalized, knowledge economies may be re-commodifying because they “demand flexibility and create insecurity” [31]. As market incomes and employment become more unstable and polarized, low- and middle-class earners increasingly stand “under pressure” [37]. A polarized labour market, defined by a growing gap between the highly skilled and highly paid who benefit from economic change and those at the bottom who do not, may be related to stress and poor health [11]. While the welfare state brings social protection associated with public health, a polarized labour market engenders insecurity and is associated, over time, with negative health consequences.

This relationship between labour market polarization and health has not been documented previously and it is therefore not easy to identify the exact mechanisms at play. One can think of a few plausible pathways. On one hand, polarization can be seen as an indicator of a less regulated labour market that does not prevent the creation of precarious, poorly paid, and stressful jobs, and probably makes it difficult for people with unsatisfactory working conditions to leave their jobs, at the risk of their own health [38]. On the other hand, the rise of top market incomes also reflects the political weight of the rich, who are less likely to favour universal, generous, and healthy social programs [39].

A welfare state that decommodifies social relations should mitigate the impact of globalization and technical change and prevent or moderate labour market polarization [31, 40]. Decommodification should also reduce the incidence of labour market risks. The welfare state works largely as an insurance mechanism against social risks such as employment termination, the loss of a spouse, sickness, disability, retirement, childbirth, or poverty [18]. We know from previous research that generous social policies can counter the consequences of adverse life events [41] and that large income losses associated with unemployment or economic hardships reduce population health [6, 7, 20, 22, 23, 29]. Welfare state decommodification is associated with lower labour market polarization and with a reduction of risk incidence, which in turn are related to population health. We thus propose the following hypotheses:

H_*2*_: *Welfare state generosity is associated with lower labour market polarization and a lower risk incidence*

H_*3a*_: *Labour market polarization is associated with higher death rates*

H_*3b*_: *The reduction of risk incidence is associated with lower death rates*

In sum, we argue that one well-known outcome of generous welfare states, decommodification, is associated with public health directly, as it provides better social protection, and indirectly, as it favours working and living conditions that make for better, more secure, and healthier lives. The common trait shared by these two outcomes of decommodification is less the overall redistribution of incomes than the level of protection and security citizens obtain against social risks.

### Data

Our dependent variable is the age-standardized death rate (from all causes) per 100 000 inhabitants, for both men and women, extracted from the OECD Health Statistics. Mortality is selected as an indicator because it is one of the most important measures of population health in comparative studies [5, 26]. In the Supporting information section, we present similar results with life expectancy at birth and at 65 years old.

The measurement of decommodification is subject to several debates and critiques. Many studies on the social determinants of health assess decommodification with welfare regime typologies contrasting three or four ideal typical regimes. This approach has the advantage of including difficult to measure regime-specific institutions [24]. Studies comparing health outcomes by regime types, however, are less likely to find significant relationships than those relying on time-variant measures [42], because regimes are time-invariant black boxes including several distinct policies [4, 33].

Others have focused on levels of public social spending, assuming that “the bigger-the better” [25, 27, 33]. Expenditures, however, are not always a good measure of decommodification as they fail to distinguish between the redistributive objectives and coverage of different programs [12, 31]. Expressed as a proportion of GDP, spending levels are also subject to fluctuations in the denominator unrelated to policy changes.

To improve upon Esping-Andersen’s early attempt at designing a measure of decommodification going beyond expenditures, Scruggs, Jahn, and Kuitto [16] constructed a welfare state generosity index that has become the standard measure of decommodification. This index is based on the generosity of three social programs addressing traditional social risks: loss of income due to sickness, unemployment, and old age. For sickness and unemployment insurance, the indices combine the income replacement rate for a typical person earning 100% of a country’s average income, the qualification period required to obtain the social benefit, the percentage of the population covered by this public insurance, and the duration and waiting period to receive the social benefit. The components for pensions include standard benefit replacement rates, standard qualification years, ratio of employer to employee pension contributions, and the coverage rates. Distinct measures thus cover sickness insurance, unemployment benefits, and pensions: we analyze their relationship to health when combined in an overall welfare state generosity index or when taken separately. A more detailed explanation of the construction of the generosity index is presented in the Supporting information section.

While we are convinced that the generosity index is the best available measure of decommodification, this index is not without shortcomings. It covers only parts of the welfare state, focusing on income protection rather than services and excluding, for example, family policies or social assistance. Moreover, the index is based on a specific group of the population: workers earning the country’s average wage. This focus led to a feminist critique of the decommodification index. Several post-industrial social programs aim instead to “re-commodify” women’s lives by favoring their participation to the labour market [30]. Aware of these critiques, we compare the effect of welfare state generosity on both men’s and women’s health.

Hypothesis 1b proposes that welfare state generosity has a direct association to population health that is not conditional on income redistribution and inequality. The Gini index for disposable income (after taxes and transfers), the most common indicator of income inequality, is used to measure income inequality. Following the standard approach, we calculate the size of redistribution as the Gini index before taxes and transfers (market Gini) minus the disposable income Gini (after taxes and services), divided by market Gini ([market Gini—disposable Gini] / market Gini). Note that we are interested in the total redistributive effect of the welfare state and that we recognize that specific programs, such as unemployment insurance, may have a redistributive effect on their own and be associated with population health.

It is not possible, within the confines of a single article on population health, to account satisfactorily for labour market polarization, a complex phenomenon with numerous determinants. The Gini index is not a good indicator for such polarization since it is more sensitive to changes in the middle of the income distribution and less affected by trends at the extremes (poor and rich) [43]. Labour market polarization and income inequality in the middle of the distribution are two distinct phenomenon driven by different factors [44]. To test hypotheses 2 and 3 about labour market polarization, we follow common practice and use the p90p10 income ratio, which is the ratio of market income between the top and bottom 10% of the distribution, before taxes and transfers [45]. To ensure that the relationship for labour market polarization does not simply reflect the association between the overall distribution of market income and health, we control for the market income Gini, which captures inequality in incomes from labour, capital, and private transfers (the results are presented in the Supporting information section).

To assess risk reduction, we use Jacob Hacker and Philipp Rehm’s new measure of the welfare state insurance function which has never been applied in studies of population health [18]. Their measure of risk reduction is based on panel surveys tracking household finances over time to calculate the importance of risk incidence mitigation by the welfare state. This measure is defined as the degree to which taxes and transfers reduce the frequency and severity of major losses in household income. Hacker and Rehm calculate the number of working age people who experience a 25% loss in market income but were insufficiently buffered by taxes and transfers to prevent a loss of 25% of disposable income, divided by the total number of working age people who experience a 25% loss in market income. In the Supporting information section, we replicate the models with a threshold of risk incidence set at losses of 50% of income.

We control for the percentage of the population aged 65 and over, the unemployment rate, and GDP per capita, three factors that are potential confounders between welfare state generosity, income distribution, or population health. An aging population and high unemployment rates are related to poorer health, increased social spending and to fiscal stress that may be associated to welfare state generosity, whereas GDP per capita decreases fiscal stress and could be related to population health. We also control for average per capita alcohol consumption, a good indicator of lifestyle habits that influence health and varies across countries and over time. Our dataset is primarily constrained by the availability of the generosity index, and it includes 21 OECD countries from 1971 to 2010. The dataset gets even smaller when considering risk reduction, a measure available for a shorter period. The descriptive statistics, the list of countries, temporal coverage, and the sources for the different variables are presented in the Supporting Information section.

### Models and methods

Our models are based on Eq 1, which is an autoregressive distributed lag equation:

$$Y_{it}=a_{0it}+{a_{1}Y}_{it-1}+{b_{2}X}_{it-5}+{b_{4}Z}_{it-5}+\varphi_{i}+\tau_{it}+\varepsilon_{it}$$ (1)

*Y* is the death rate at time *t* in country *i*. It is predicted by a constant *a*, a lagged dependent variable *Y*_t-1_, the independent variable X_t-5_ (either the generosity index or measures of redistribution, labour market polarization, or risk reduction), a vector of controls Z_t-5_, a country fixed effect *φ_i_*, a country-specific time trend *τ_it_* an error term *ε_it_*. We use panel corrected standard errors to correct for heteroskedasticity in the error term, as well as an AR1 correction for serial correlation in the error term [46].

Several statistical considerations justify this choice of model. The strong serial correlation in the dependent variable militates for the inclusion of a lagged dependent variable (LDV). However, the inclusion of the LDV represents a trade-off between omitted variable bias and overfitting, since the lagged dependent variable explains a large proportion of the variance. While similar macro-level studies in population health are not using LDVs [2, 25, 47], in political science and economics, it is common to prevent omitted variable biases by using LDVs [48]. We thus present models with LDVs in the main manuscript and without a LDV in the Supporting information section.

We tested different lag structures and found that the relationship between our variables of interest and population health tends to take five years to unfold. Therefore, in the main analysis, we present models with five years lags (see [47] for a similar modelling strategy). These models assume there is no immediate relationship between X and Y. In the Supporting information section, we supplement the analysis with models including a one year and a five-year lag.

Our models include country fixed effects. These are technically justified by a Hausman test but also theoretically warranted since we cannot control for several unobserved factors influencing the health of a population. By including country fixed effects, our equations analyze the relationship between an independent variable and the dependent variable within a country. To model the negative, but country-specific, trend in age-standardized death rates, we include a country-specific time trend. Using fixed effects, country-specific time trends and LDVs to identify the relations with a slowly moving dependent variable is a very conservative modelling strategy, that purposely makes it difficult to identify significant relationships.

We run stationarity tests on each variable. They show that the generosity index, the p90p10 ratio, and the risk reduction variable are stationary, while the dependent variables are stationary around a trend. The disposable income Gini and the control variables (except the unemployment rate) are non-stationary, whereas the tests are uncertain for the redistribution measure. Regressing a non-stationary variable on a stationary series biases the equation [49]. We thus first difference the disposable income Gini and the control variables, except for the unemployment rate, to make them stationary [50]. We choose to present the redistribution measure in levels, to allow for a more meaningful comparison with the effect of risk reduction and the p90p10 ratio, but present it in first difference in the Supporting information section in case it would be non-stationary.

The next section presents the results. It starts with a test of H_2,_ associating the generosity index to risk reduction or labour market polarization. Then, we assess H_1a_ and H_1b_ by regressing welfare state generosity on the age standardized death rate following equation 1. Also using equation 1, we then test *H*_*3a*_ and *H*_*3b*_ by regressing either risk reduction, labour market polarization, disposable income inequality or income redistribution to predict the death rate. From these models, long-term relationships can be calculated, to represent the cumulative association of a change in the independent variable on the dependent variable as T reaches ∞. Their coefficient is determined by the following formula *b*_1_+*b*_2_/1-*a*_1_, while their standard error is calculated with the Bewley transformation [50]. Finally, to consider H_1c_ (welfare state generosity is associated with lower death rates even when controlling for income redistribution), we present additional models including measures of income redistribution and generosity together in the same equation. We control the income redistribution at T-5 and T-1 allowing for the association of the generosity index and the death rate to be mediated by generosity’s association with the income redistribution over time. We also present models confirming that the generosity index maintains an association with mortality, even when controlling for the p90p10 ratio and for risk reduction.

## Results

**Fig 1:**
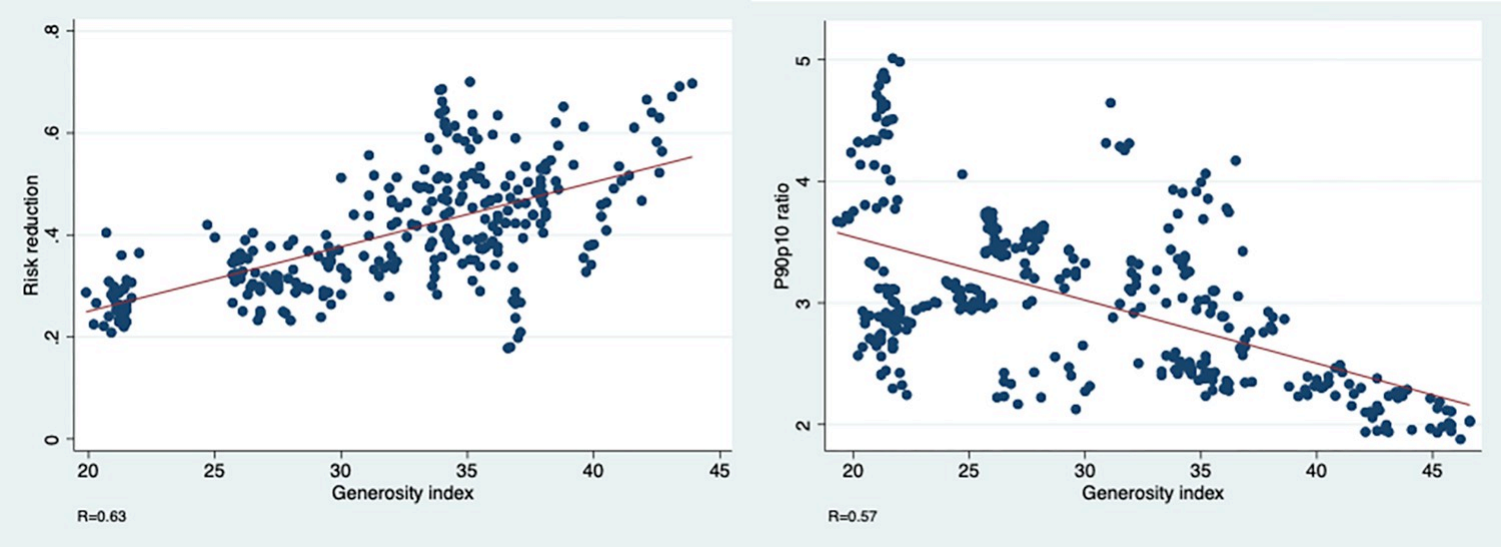
Relationship between generosity, risk reduction indices and the p90p10 ratio.

We first assess the relationship between our main variables to test H_2_. Fig 1 displays a clear bivariate correlation between the generosity index, on one hand, and risk reduction or labour market polarization on the other. To save space, we present formal regression models between these three variables in the Supporting information section, using additional controls. They reveal that higher generosity is associated with lower labour market polarization and that countries with more generous welfare states better mitigate labour markets risks.

Table 1 presents the negative associations between the generosity index and the age-standardized death rate. The death rate of both men and women is lower when the generosity index increases. Of the three components of the generosity index, pension generosity has the strongest relationship.

**Table 1:** Relationships between the generosity index and the age-standardized death rate, with country fixed effects and country specific time trends.

	(1)	(2)	(3)	(4)	(5)	(6)	(7)	(8)
	Women	Men	Women	Men	Women	Men	Women	Men
Lagged dependent variable	0.769***	0.738***	0.753***	0.724***	0.731***	0.728***	0.745***	0.722***
	(0.0318)	(0.0277)	(0.0307)	(0.0342)	(0.0318)	(0.0273)	(0.0311)	(0.0345)
Generosity T-5	-1.038***	-1.072**						
	(0.394)	(0.440)						
Unemployment gen. T-5			-0.385	-0.760				
			(0.926)	(1.139)				
Pensions gen T-5					-3.931***	-3.291***		
					(0.829)	(0.915)		
Sickness gen. T-5							-1.149	-0.860
							(0.863)	(1.210)
Δ GDP/cap. T-5	-0.000822	-0.00112	-0.00115	-0.00129	-0.00118	-0.00131	-0.00106	-0.00123
	(0.00127)	(0.00146)	(0.00152)	(0.00218)	(0.00128)	(0.00146)	(0.00152)	(0.00218)
Δ alcool T-5	-3.232	-5.122**	-0.923	-2.167	-1.698	-3.689	-1.176	-2.468
	(2.069)	(2.561)	(1.774)	(2.517)	(1.956)	(2.450)	(1.772)	(2.519)
Unemployment rate T-5	0.186	0.290	0.136	0.369	0.420	0.641*	0.0798	0.351
	(0.320)	(0.381)	(0.390)	(0.511)	(0.312)	(0.365)	(0.400)	(0.513)
Δ pop. 65+	9.019	19.99***	6.459	13.30	8.482	20.07***	6.864	14.22*
	(7.061)	(7.602)	(6.854)	(8.381)	(7.142)	(7.647)	(6.938)	(8.416)
Constant	5,489***	12,625***	5,909***	13,231***	6,432***	13,099***	6,128***	13,356***
	(927.2)	(1,535)	(903.7)	(1,860)	(929.6)	(1,519)	(914.0)	(1,875)
Observations	703	703	749	749	711	711	748	748
R-squared	0.988	0.992	0.988	0.992	0.987	0.991	0.988	0.992
Number of countries	20	20	20	20	20	20	20	20

Standard errors in parentheses.

*** p<0.01

** p<0.05

* p<0.1.

Table 2 presents the association between four variables that may help to explain how welfare state generosity relates to population health: disposable income inequality, income redistribution, labour market polarization, and risk reduction. The association between the p90p10 ratio and the death rate is particularly clear: a more unequal distribution of market income measured by the share of income allocated to the richest decile relative to the poorest decile is correlated positively and significantly with the age-standardized death rate, for both men and women. Interestingly, risk reduction is correlated negatively with the death rates for men, but not for women. In contrast, our indicators of income redistribution—the Gini for disposable income and the relative redistribution measure—are not associated significantly with death rates.

**Table 2:** Relationships between disposable income inequality, income redistribution, labour market polarization and risk reduction and the age-standardized death rate, with country fixed effects and country specific time trends.

	1	2	3	4	5	6	7	8
	Women	Men	Women	Men	Women	Men	Women	Men
Lagged dependent variable	0.706***	0.544***	0.493***	0.651***	0.753***	0.716***	0.761***	0.726***
	(0.0362)	(0.0452)	(0.0847)	(0.100)	(0.0282)	(0.0340)	(0.0273)	(0.0336)
P90p10 T-5	19.47***	41.57***						
	(5.942)	(9.925)						
Risk reduction T-5			-32.27	-82.52**				
			(22.63)	(35.11)				
Δ Gini disp T-5					-2.214	0.592		
					(1.886)	(2.567)		
Redis. T-5							-14.04	-19.10
							(36.32)	(56.58)
Δ GDP/cap. T-5	-0.00111	-0.00178	0.00186	0.00280	-0.000849	-0.000685	-0.000853	-0.000681
	(0.00127)	(0.00155)	(0.00211)	(0.00286)	(0.00149)	(0.00218)	(0.00150)	(0.00218)
Δ alcool T-5	0.498	-0.428	1.078	-1.840	0.685	0.490	0.616	0.297
	(2.229)	(3.304)	(2.755)	(4.004)	(1.765)	(2.490)	(1.747)	(2.444)
Unemployment rate T-5	0.314	0.374	3.619***	3.807***	0.281	0.393	0.175	0.418
	(0.341)	(0.439)	(0.834)	(1.165)	(0.367)	(0.509)	(0.366)	(0.506)
Δ pop. 65+	3.010	14.69	-20.26**	-14.63	-0.373	6.589	-0.371	5.004
	(6.648)	(9.704)	(9.631)	(13.41)	(6.589)	(8.114)	(6.648)	(8.215)
Constant	7,534***	23,305***	3,557	3,552	6,332***	14,438***	6,186***	14,144***
	(1,037)	(2,391)	(2,207)	(3,698)	(893.8)	(1,945)	(879.5)	(1,932)
Observations	416	416	301	301	737	737	745	745
R-squared	0.990	0.992	0.978	0.985	0.989	0.991	0.989	0.992
Number of countries	20	20	18	18	20	20	20	20

Standard errors in parentheses.

*** p<0.01

** p<0.05

* p<0.1.

**Fig 2:**
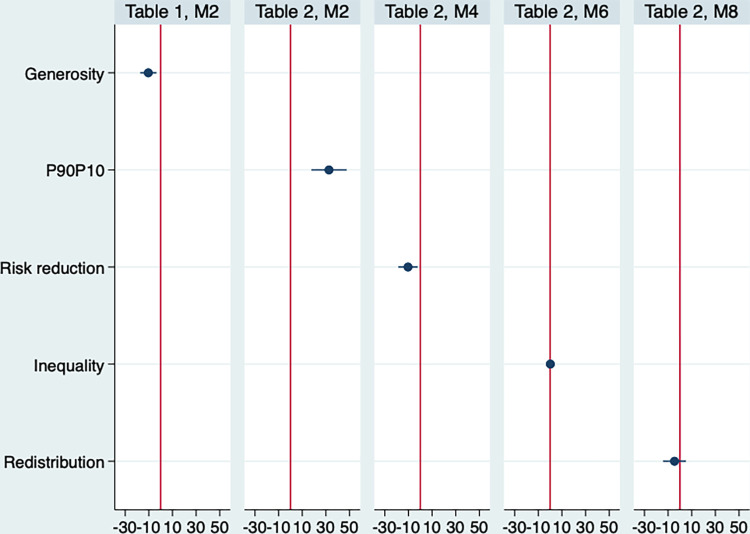
Standardized coefficients of the main variables on men’s death rates, based on models from Tables 1 and 2.

Fig 2 presents a graphical representation of the effect of the main variables in the models of Tables 1 and 2 on men’s mortality. It shows standardized coefficients: a one-unit change represents a standard deviation. Risk reduction and welfare state generosity significantly reduce men’s mortality, while the p90p10 ratio increases it and the effect of inequality and redistribution is not different from 0.

**Fig 3:**
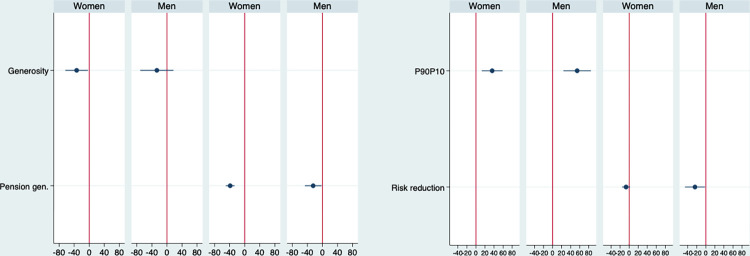
Long run multipliers of the standardized coefficients of the main variables on death rates, based on models from Tables 1 and 2.

Fig 3 displays the long-run multipliers of the standardized coefficients of the generosity index, the pension generosity, the p90p10 ration and the risk reduction measures, which are the variables that are significant in Tables 1 and 2, based on their respective models in presented in these two tables. It reveals that in the long term, a one standard deviation increase in the generosity index reduces women’s age standardized death rate by about 33 but has no effect on men, that an increase in the p90p10 increases men’s mortality by 54 and women’s by 36. Long-run multipliers are not significant for redistribution or the disposable Gini (not shown) whereas the reduction of risk incidence has a significant negative long-term association with death rates, as it reduces men’s mortality by 24 but has no effect on women.

Table 3 shows that the generosity index maintains its association with population health, even when controlling for redistribution and inequality. The variance inflation factors suggest that multicollinearity is not an issue, since the highest VIF (for redistribution) is 3.34, whereas the others are around 1 or 2. Models 1 to 4 reveal that not only does the generosity index keeps its significance when controlling for redistribution and for disposable income Gini, but its coefficient does not become smaller than in Table 1, suggesting that the relation between the generosity index and the death rate is not mediated by these two variables. Models 5 to 8 indicate that generosity maintains its association with population health even when risk reduction and the p90p10 ratios are included in the models, except in model 6, where the association between the generosity index and men’s health become insignificant when p90p10 is included, suggesting that some of the relationships are mediated [51]. The time-series cross-sectional nature of our data, using observational variables that are strongly autocorrelated, prevent us from introducing more sophisticated mediation models.

**Table 3:** Relationships between the generosity index and the age-standardized death rate, controlling for Gini, redistribution, p90p10, and risk reduction, and including fixed effects and country specific time trends.

	1	2	3	4	5	6	7	8
	Women	Men	Women	Men	Women	Men	Women	Men
Lagged dependent variable	0.759***	0.716***	0.755***	0.728***	0.686***	0.478***	0.484***	0.577***
	(0.0323)	(0.0290)	(0.0316)	(0.0281)	(0.0395)	(0.0492)	(0.0793)	(0.0980)
Generosity t-5	-1.304***	-1.744***	-1.947***	-1.989***	-1.232***	-1.034	-4.479***	-5.647**
	(0.390)	(0.447)	(0.427)	(0.477)	(0.468)	(0.800)	(1.581)	(2.665)
Δ Gini disp T-1	-2.425	-0.354						
	(1.927)	(2.384)						
Δ Gini disp T-5	-1.389	2.020						
	(1.726)	(2.049)						
Redis T-1			71.80	-67.43				
			(50.42)	(63.19)				
Redis. T-5			73.92*	127.9**				
			(43.25)	(58.42)				
p90p10 t-1					1.970	-4.786		
					(8.397)	(11.79)		
P90p10 T-5					15.86**	40.53***		
					(6.819)	(11.23)		
Risk reduction T-1							-20.13	-63.81**
							(20.51)	(30.28)
Risk reduction T-5							-23.15	-96.37***
							(23.39)	(35.04)
Δ GDP/cap. T-5	-0.000539	-0.000746	-0.000368	-0.000716	-0.00129	-0.00255	0.00377*	0.00364
	(0.00127)	(0.00146)	(0.00130)	(0.00147)	(0.00127)	(0.00155)	(0.00213)	(0.00238)
Δ alcool T-5	-3.005	-4.474*	-2.780	-4.780*	0.572	-0.759	-0.935	-3.663
	(2.038)	(2.536)	(2.025)	(2.465)	(2.375)	(3.401)	(2.245)	(3.377)
Unemployment rate T-5	0.327	0.272	0.282	0.304	0.436	0.562	3.742***	4.143***
	(0.326)	(0.386)	(0.331)	(0.380)	(0.355)	(0.479)	(0.877)	(0.985)
Δ pop. 65+	7.275	17.14**	7.474	16.36**	-3.958	7.157	-21.78**	-17.10
	(6.889)	(7.717)	(7.052)	(7.736)	(7.924)	(11.37)	(10.10)	(11.58)
Constant	5,794***	13,832***	5,846***	13,190***	8,083***	26,838***	3,301	6,395
	(937.9)	(1,606)	(920.8)	(1,561)	(1,163)	(2,748)	(2,818)	(4,462)
Observations	665	665	671	671	399	399	278	278
R-squared	0.988	0.991	0.989	0.992	0.989	0.992	0.981	0.986
Number of countries	20	20	20	20	20	20	18	18

Standard errors in parentheses.

*** p<0.01

** p<0.05

* p<0.1.

We conducted several robustness checks, none of which altered the main results. We used a jackknife technique removing one country at a time to ensure that the results were not driven by a particular country. Second, in the Supporting information section, we present models without control variables to ensure that our results are not model dependent. Third, vector autoregression models have been conducted to ensure that reverse causality between the generosity index and the age-standardized death rate is not an issue. Fourth, since the series of the Gini indices are longer than the p90p10 ratio series, we replicated the analyses by restricting models 5 to 8 to the same country and years as in models 1 and 4 in Table 2. The association between the Gini index, redistribution and health generally remains insignificant in these additional analyses. Fifth, we included poverty rates as an additional measure of redistribution. Sixth, the Supporting information section also displays replications of Tables 1 and 2 for life expectancy at birth and at 65 years old, revealing that our results are similar for other measures of population health. Seventh, we replicate Tables 1 to 3 without lagged dependent variables and by expressing inequality in levels and redistribution in first difference. This allows us to confirm that the null effects of redistribution and disposable income inequality on health is not explained by first differenced variables. In few models, redistribution is associated with a reduced death rate, but the results are inconsistent across models, while disposable income inequality and poverty rates never relate to the death rate. Eight, we control for public health care spending as a proportion of GDP and the share of private health care spending to ensure that more encompassing health care services, which are positively correlated with generosity, are not a confounding factor of the relationship between generosity and population health. Finally, our measure of welfare state generosity being mostly about income protection, we replicate the main models with a measure of total social expenditures expressed as a proportion of GDP, which also includes services. It doesn’t alter the effect of generosity and of the Gini coefficient, redistribution, p90p10 and the risk reduction measure. Public expenditures alone are not significantly related to population health.

## Discussion and conclusion

Our main contribution is to highlight the direct and indirect relationships between decommodification and population health. It is through decommodification, and not merely redistribution, that the welfare state promotes health, in two ways. Social programs decommodify social relations and provide better social protection, which is associated with healthier lives. This result confirms hypothesis 1_a._ This result is not entirely new. We knew, for instance, that there was an association between welfare state generosity and population health [1, 2, 26, 52]. We also found that of the three components of the generosity index, pension has the strongest effect, a result similar to Beckfield and Bambra [2]. Comparative work on the relationship between the institutional structure of pension regimes and health in old age suggests indeed that basic, decommodifying pensions are strongly associated with health [14, 35]. We clarified within an integrated framework based on the concept of decommodification and attentive to gender, the respective relationships between welfare state generosity, redistribution, labour market polarization, and risk reduction.

The gender differences proposed by hypothesis 1_b_ are not confirmed by our results. The results presented in Fig 3 suggest that for men a change in welfare state generosity is associated with the death rate after five years, but the relationship does not accumulate overtime to become larger than the coefficients presented in Table 1. However, the short-term coefficient of the generosity index on men’s health falls in between the large confidence intervals of the coefficient of the long-run multiplier for women’s health. We therefore cannot confirm hypothesis 1_b_ to conclude that there is a statistically significant difference in the coefficient of the generosity index for men and women’s health.

We have shown that the welfare state decommodifies the labour market, bringing about less polarized market incomes and reducing the incidence of labour market risks, two factors associated with lower premature mortality, for men in particular. Indeed, Fig 1 and models presented in the Supporting information section reveal a significant associated between the generosity index and the p90p10 ratio or the risk reduction measure, allowing us to reject the null of H_2_. This confirms the relationship between welfare state generosity and risk reduction presented by Hacker and Rehm [18] (see also [41]). Tables 2 and 3 showed significant associations between the p90p10 ratio or the risk reduction measure and the age-standardized death rates, allowing us to reject the null for hypothesis 3.

That risk reduction only relates to men’s health may be explained by women’s apparently stronger resilience than men in the face of socioeconomic adversity [53, 54]. It could also be explained by the fact that the Hacker-Rehm risk reduction measure relies on household income which may obscure important variations within households between men and women. One would need a more individualized measure of risk reduction to compare how it relates to men and women’s health. More research needs to be done on the different relationships between risk reduction and men and women’s health, as this is the first study of population health to use this variable.

Welfare state generosity’s positive association with population health is therefore not conditional on the well-established association between the welfare state and income redistribution. While the generosity index, the p90p10 ratio, and the risk reduction measures have significant long- and short-term relationships, income redistribution, measured by disposable income inequality or the size of redistribution, does not have a significant relationship with the health of populations. The security and protection against social risks provided by social policies appears more important for health than their egalitarian consequences. What relates to health is less the redistribution of income than the lower vulnerability to risks and social insecurity associated with welfare state generosity. It is not because unemployment insurance is inherently redistributive that it relates to better population health [4, 6, 29]. It is because it reduces the incidence of labor market risks and mitigate their impact on population health. At the macro level, it is the reduction of labour market risks that matters the most for population health, not the redistribution of income.

As we conclude, it is worth mentioning some limitations of this study. We mainly use the p-value to determine whether the correlations are significant. It is still possible that variables that do not have a statistically significant relationship may have a statistically significant association in an alternative model. Similarly, we cannot demonstrate a causal effect of our main variables since this is a macro-level study with observed data, without an experimental design. This type of design also prevents us from using more sophisticated mediation models.

It is not possible, within the confines of a comparative study of broad institutional factors, to identify the exact mechanisms linking social arrangements to population health. While we found that decommodification is associated with population health, even if we control for risk reduction, labor market polarization and income redistribution, we could not identify the drivers of this direct relationship. It is possible that high decommodification is associated with better social protection and better public services that reduce stress and improve individuals’ sense of autonomy, security, and health. To identify the mechanisms at play, one would need to pursue more fine-grained inquiries about the consequences of specific public measures (see for example [4, 8, 29]). This study nevertheless highlights the relevance of welfare state institutions for public health and opens new avenues for future research. To our knowledge, this article is the first to highlight the relationship between labour market polarization and population health and to include a measure of risk reduction in studies of the relationships between the welfare state and health. Future research could also aim to identify better the causal mechanism linking a polarized labour market to poor health.

## Supporting information

S1 File (ZIP)
