# Supplementary material for: Welfare state decommodification and population health
Source: PLoS One. 2022 Aug 31;17(8):e0272698. doi: 10.1371/journal.pone.0272698 (PMC9432727; doi:10.1371/journal.pone.0272698)
Supplement: S1 File — (ZIP) [file pone.0272698.s001.zip › Table A1. Descriptive statistics.docx]

**Descriptive statistics**

The study focuses on 21 OECD countries: Australia, Austria, Belgium, Canada, Denmark, Finland, France, Germany, Greece, Ireland, Italy, Japan, Netherlands, New Zealand, Norway, Portugal, Spain, Sweden, Switzerland, UK, USA.

Note that the risk-reduction measure is available mostly from 1994 to 2014, but the series is longer in some countries (like Germany, the UK and the USA). We used within country linear interpolation to fill missing data in several European countries between 2001 to 2004.

The p90p10 measure becomes widely available in the late 1990s.

The generosity index is limited to 1971 to 2010.

The other measures are generally available from 1970 to 2017.

## Table A1. Descriptive statistics

| Variable | Obs | Mean | Std.Dev. | Min | Max | Reference |
| --- | --- | --- | --- | --- | --- | --- |
| Death rate F | 1154 | 934.48 | 260.338 | 425.7 | 1673.3 | OECD Health Statistics |
| Death rate M | 1154 | 1415.003 | 361.71 | 756.5 | 2374.1 |  |
| Generosity index | 755 | 31.188 | 6.983 | 10.8 | 46.6 | Scruggs et al. 2017. Welfare Entitlements Dataset |
| Pension generosity | 766 | 11.562 | 2.143 | 6.4 | 17.1 |  |
| Sickness generosity | 816 | 10.038 | 4.227 | 0 | 18.2 |  |
| Unemployment generosity | 817 | 9.496 | 2.726 | 1.7 | 14.5 |  |
| P90p10 | 543 | 3.022 | .681 | 1.878 | 5.223 | Brady et al., 2020. *Comparative welfare state dataset* |
| Risk reduction, 25% threshold | 383 | 0.407 | .117 | 0.147 | 0.702 | Hacker and Rehm, 2020 |
| Risk reduction, 50% threshold | 383 | 0.547 | 0.127 | 0.26 | 0.841 |  |
| Disposable Gini | 1005 | 28.871 | 4.008 | 20.2 | 38.2 | Solt, 2020. SWIID |
| Market Gini | 1005 | 45.425 | 4.184 | 35.5 | 54 |  |
| Poverty 50 | 579 | .098 | .037 | .027 | .179 |  |
| Δ GDP/capita | 1022 | 557.061 | 842.037 | -3946.844 | 12023.05 | OECD *National accounts* |
| Δ alcohol consumption | 1095 | -.002 | .544 | -4.3 | 5.9 | OECD *Health statistics* |
| Unemployment rate | 1184 | 5.982 | 4.128 | 0 | 27.5 | Armingeon et al., 2020. *Comparative Political Dataset* |
| Δ pop 65+ | 1164 | .164 | .163 | -.6 | 1.08 |  |
|  | | | | | |  |
