# Supplementary material for: Welfare state decommodification and population health
Source: PLoS One. 2022 Aug 31;17(8):e0272698. doi: 10.1371/journal.pone.0272698 (PMC9432727; doi:10.1371/journal.pone.0272698)
Supplement: S1 File — (ZIP) [file pone.0272698.s001.zip › Table A14. Inclusion of public social expenditures in the main models..docx]

## Table A14. Inclusion of public social expenditures in the main models.

|  | (1) | (2) | (3) | (4) | (5) | (6) | (7) | (8) |
| --- | --- | --- | --- | --- | --- | --- | --- | --- |
|  | Women | Men | Women | Men | Men | Men | Men | Men |
|  |  |  |  |  |  |  |  |  |
| Lagged dependent variable | 0.682*** | 0.619*** | 0.654*** | 0.542*** | 0.535*** | 0.537*** | 0.375*** | 0.549*** |
|  | (0.0405) | (0.0460) | (0.0460) | (0.0442) | (0.0445) | (0.0442) | (0.0642) | (0.120) |
| Public social expenditures t-5 | 0.510 | 0.909 | 1.794*** | 3.054*** | 2.986*** | 2.853*** | 3.428*** | 2.457 |
|  | (0.497) | (0.682) | (0.610) | (0.737) | (0.721) | (0.732) | (1.113) | (2.295) |
| Generosity T-5 |  |  | -3.779*** | -6.596*** | -6.999*** | -7.222*** | -5.236*** | -6.561*** |
|  |  |  | (0.540) | (0.849) | (0.850) | (0.865) | (1.420) | (2.443) |
| Δ Gini disp T-5 |  |  |  |  | 1.759 |  |  |  |
|  |  |  |  |  | (2.705) |  |  |  |
| Redis. T-5 |  |  |  |  |  | 104.6 |  |  |
|  |  |  |  |  |  | (81.03) |  |  |
| P90p10 T-5 |  |  |  |  |  |  | 47.89*** |  |
|  |  |  |  |  |  |  | (14.50) |  |
| Risk reduction T-5 |  |  |  |  |  |  |  | -72.73* |
|  |  |  |  |  |  |  |  | (38.21) |
| Δ GDP/cap. T-5 | 0.000192 | 0.000481 | 0.000600 | 0.00105 | 0.00127 | 0.00124 | 0.000213 | 0.00450 |
|  | (0.00140) | (0.00199) | (0.00150) | (0.00170) | (0.00170) | (0.00170) | (0.00181) | (0.00289) |
| Δ alcool T-5 | -2.402 | -4.053 | -1.739 | -3.504 | -4.613 | -4.435 | -2.810 | -4.412 |
|  | (1.936) | (2.567) | (2.375) | (2.822) | (2.814) | (2.775) | (3.683) | (4.128) |
| Unemployment rate T-5 | -0.247 | -0.343 | -0.925* | -1.571** | -1.287** | -1.265** | -0.688 | 2.766** |
|  | (0.467) | (0.687) | (0.476) | (0.628) | (0.620) | (0.603) | (0.756) | (1.318) |
| Δ pop. 65+ | 7.767 | 17.14** | 12.49* | 27.36*** | 24.86*** | 25.21*** | 21.46* | -11.01 |
|  | (6.885) | (8.300) | (7.535) | (8.665) | (8.476) | (8.491) | (12.76) | (13.93) |
| Constant | 7,335*** | 17,454*** | 8,929*** | 23,022*** | 23,256*** | 23,180*** | 32,749*** | 6,507 |
|  | (1,141) | (2,374) | (1,371) | (2,412) | (2,427) | (2,441) | (3,565) | (4,756) |
|  |  |  |  |  |  |  |  |  |
| Observations | 606 | 606 | 571 | 571 | 567 | 568 | 366 | 285 |
| R-squared | 0.985 | 0.988 | 0.985 | 0.988 | 0.988 | 0.988 | 0.990 | 0.985 |
| Number of ctyid | 20 | 20 | 20 | 20 | 20 | 20 | 20 | 18 |
| Standard errors in parentheses | | |  |  |  |  |  |  |
| *** p<0.01, ** p<0.05, * p<0.1 | | |  |  |  |  |  |  |
