# Supplementary material for: Welfare state decommodification and population health
Source: PLoS One. 2022 Aug 31;17(8):e0272698. doi: 10.1371/journal.pone.0272698 (PMC9432727; doi:10.1371/journal.pone.0272698)
Supplement: S1 File — (ZIP) [file pone.0272698.s001.zip › Table A13. Models predicting the p90p10 ratio and the risk-reduction measure .docx]

Table A13 demonstrates that the generosity index is negatively correlated with labour market polarization, indicating that not only does welfare state generosity directly influences population health, but it also has an indirect relationship, through the labour market. In models 1 to 3, we add three variables that, according to the literature, could influence the distribution of market income. First, we include public investment in education as a proportion of GDP, a factor that could affect inequality by influencing human capital distribution (Goldin and Katz, 2008). We also control for two measures related to the labour market, namely union density and corporatism, as measured by the corporatism index produced by Detlef Jahn (2016). Union density compresses wages, particularly at the bottom and top of the income distribution (Huber, Huo, and Stephens, 2019), whereas corporatism is associated with lower inequality (Pontusson, 2005). Our models also incorporate the same controls as for the previous analyses since these are standard control variables for models that predict the level of inequality, to which we add deindustrialization and economic openness, two variables that could influence the provision of social policies and the level of inequality (Wren, 2013).

Models 1 to 3 follow the same specifications as the main models, with a lagged dependent variable, fixed effects, a country-specific time trend, and panel corrected standard errors. Inequality studies assume that the effect on income distribution of a change in public policy is relatively rapid, so we do not model the independent variables with a five-year lag. Instead, in the models presented in Table A13, we lag the independent variables by one year (models 1 and 2) and present an unrestricted model (3) with variables at T-0 and T-1. Table A13 shows that the generosity index has a significant negative association with the p90p10 ratio.

Table A13. Models predicting the p90p10 ratio and the risk-reduction measure

|  |  |  |  |  |  |  |  |
| --- | --- | --- | --- | --- | --- | --- | --- |
|  | (1) | (2) | (3) | (4) | (5) | (6) | (7) |
|  | ip90p10 | ip90p10 | ip90p10 | riskred25 | riskred25 | riskred25 | riskred25 |
|  |  |  |  |  |  |  |  |
| Lagged dependent variable | 0.512*** | 0.517*** | 0.519*** | 0.819*** | 0.240** | 0.400*** | 0.174* |
|  | (0.0435) | (0.0515) | (0.0618) | (0.0492) | (0.0963) | (0.0835) | (0.0982) |
| Generosity T-0 |  |  | -0.00138 |  |  | 0.00841* | 0.0118*** |
|  |  |  | (0.00515) |  |  | (0.00498) | (0.00315) |
| Generosity T-1 | -0.0114*** | -0.0145*** | -0.0132** | 0.00226*** | 0.00713 | -0.00957** | 0.00402 |
|  | (0.00338) | (0.00450) | (0.00538) | (0.000727) | (0.00472) | (0.00443) | (0.00369) |
| Public education spending t-0 |  |  | -0.0113 |  |  | 0.0335*** | 0.0455*** |
|  |  |  | (0.0229) |  |  | (0.0129) | (0.00695) |
| Public education spending t-1 |  | 0.00205 | 0.0170 |  |  | -0.00407 | 0.00692 |
|  |  | (0.0113) | (0.0213) |  |  | (0.0138) | (0.0112) |
| Union density T-0 |  |  | 0.00159 |  |  | -0.0100** | -0.0129*** |
|  |  |  | (0.00306) |  |  | (0.00464) | (0.00383) |
| Union density T-1 |  | 5.27e-05 | -0.00225 |  |  | 0.00790** | -0.00585 |
|  |  | (0.00175) | (0.00321) |  |  | (0.00402) | (0.00362) |
| Corporatism T-0 |  |  | -0.00963 |  |  | -0.00573 | -0.0141* |
|  |  |  | (0.0110) |  |  | (0.00681) | (0.00853) |
| Corporatism T-1 |  | -0.00854 | -0.00400 |  |  | 0.00728 | 0.000638 |
|  |  | (0.0114) | (0.0116) |  |  | (0.00850) | (0.0104) |
| Δ GDP/cap. T-0 |  |  | -3.30e-06 |  |  | 6.18e-07 | 3.09e-06 |
|  |  |  | (5.65e-06) |  |  | (6.43e-06) | (6.40e-06) |
| Δ GDP/cap. T-1 |  | 7.52e-06* | 5.19e-06 |  | -6.66e-06* | -1.65e-06 | 2.14e-06 |
|  |  | (4.30e-06) | (5.07e-06) |  | (3.49e-06) | (6.21e-06) | (5.66e-06) |
| Unemployment T-0 |  |  | 0.000326 |  |  | 0.00931** | 0.0123*** |
|  |  |  | (0.00478) |  |  | (0.00404) | (0.00354) |
| Unemployment T-1 |  | 0.000490 | 0.00221 |  | 0.00199 | -0.00878** | -0.00466 |
|  |  | (0.00188) | (0.00422) |  | (0.00206) | (0.00372) | (0.00374) |
| Δ pop. 65+ T-0 |  |  | -0.0331 |  |  | -0.0105 | -0.0356 |
|  |  |  | (0.0553) |  |  | (0.0207) | (0.0218) |
| L.delderly T-5 |  | -0.0173 | -0.0145 |  | -0.0196 | 0.0216 | 0.00533 |
|  |  | (0.0355) | (0.0588) |  | (0.0272) | (0.0161) | (0.0203) |
| Deindustrial. T-0 |  |  | -0.0109** |  |  | -0.00915*** | -0.00325 |
|  |  |  | (0.00471) |  |  | (0.00222) | (0.00250) |
| Deindustrial. T-1 |  | -0.00120 | 0.00475 |  | 0.000692 | 0.00311 | 0.00439* |
|  |  | (0.00264) | (0.00416) |  | (0.00194) | (0.00263) | (0.00235) |
| Trade openness T-0 |  |  | -0.00282*** |  |  | 7.62e-06 | -0.000130 |
|  |  |  | (0.000962) |  |  | (0.000827) | (0.000697) |
| Trade openness T-1 |  | -0.00136 | -2.17e-05 |  | 0.000789 | 0.000605 | 0.000555 |
|  |  | (0.000844) | (0.000885) |  | (0.000565) | (0.000781) | (0.000671) |
| Constant | -16.22*** | -16.11** | -19.53*** | 0.00161 | -6.395 | -1.150 | -13.93 |
|  | (2.497) | (6.550) | (5.708) | (0.0112) | (17.94) | (1.514) | (11.63) |
| Panel corrected standard errors | Yes | Yes | Yes | No | Yes | Yes | Yes |
| Country specific trends | Yes | Yes | Yes | No | Yes | Yes | Yes |
| Country fixed effects | Yes | Yes | Yes | No | Yes | No | Yes |
| Observations | 420 | 392 | 371 | 301 | 301 | 269 | 269 |
| R-squared | 0.989 | 0.990 | 0.990 |  | 0.878 | 0.879 | 0.903 |
| Number of ctyid | 21 | 21 | 21 | 19 | 19 | 19 | 19 |
| Standard errors in parentheses | |  |  |  |  |  |  |
| *** p<0.01, ** p<0.05, * p<0.1 | |  |  |  |  |  |  |

Models 4 to 7 test the association between the generosity index and the risk-reduction measure. Models 4 and 7 use random effects and show that welfare state generosity is positively correlated with risk reduction. Model 7 includes all the same control as to predicting labour market polarization, whereas model 4 removes controls. Models 5 and 6 include country fixed effects. They do not show a significant association between generosity and risk reduction. This may be because of the relatively short series and the slowly varying nature of risk reduction and of the generosity index. As such, while we cannot confirm that a change in generosity is association with more risk reduction within a country, the random effect models confirm that higher levels of welfare state generosity are associated with more risk reduction.
