# Supplementary material for: Welfare state decommodification and population health
Source: PLoS One. 2022 Aug 31;17(8):e0272698. doi: 10.1371/journal.pone.0272698 (PMC9432727; doi:10.1371/journal.pone.0272698)
Supplement: S1 File — (ZIP) [file pone.0272698.s001.zip › Table A12. Models with alternative measures of the income distribution .docx]

Table A12 replicates Table 2 with alternative measures of the income distribution. Models 1 to 3 use the level of the disposable income Gini rather than the first difference as in the main models, while models 4 to 6 use the first difference of redistribution. While models 3 and 6 show a negative association between higher inequality or higher redistribution and mortality (for men only), the relationship is not robust to removing the lagged dependent variables, as shown in models 1 and 4. Models 7 and 8 reveal that more Gini for market income is negatively correlated with mortality, which goes against theoretical expectations. Models 9 and 10 include a measure of poverty rates, which is the percentage of the population living with 50% or less of the median national income, after taxes and transfers. We do not find a significant association (at p=0.05) of poverty on population health, but we are not confident in this null result for several reasons, since we know from previous studies that lifting the incomes of the poor contribute to better health (Nelson and Fritzell, 2014). As a measure of poverty, we use the proportion of people living on less than 50% of a country's average income. Not only is this measure only available for a relatively short series, but it tells us relatively little about the situation of people living in poverty. Indeed, we do not measure the duration of poverty, nor the average income level of people experiencing poverty. For example, the health of the population in a country where 10% of the population lives in temporary poverty with incomes representing 40% of the average wage is likely to be better than population health in a country where the same proportion of people live in chronic poverty with incomes of barely 20% of the average income. These two countries may exhibit the same level of poverty according to our measure, revealing its limitations. Unfortunately, there is no better international measure of poverty available over a long period of time. This explains why we preferred not to include the poverty rates in the main article.

## Table A12. Models with alternative measures of the income distribution

|  |  |  |  |  |  |  |  |  |  |  |
| --- | --- | --- | --- | --- | --- | --- | --- | --- | --- | --- |
|  | 1 | 2 | 3 | 4 | 5 | 6 | 7 | 8 | 9 | 10 |
|  | Men | Women | Men | Men | Women | Men | Women | Men | Women | Men |
|  |  |  |  |  |  |  |  |  |  |  |
| Lagged dependent variable |  | 0.651*** | 0.610*** |  | 0.645*** | 0.601*** | 0.635*** | 0.597*** | 0.573*** | 0.582*** |
|  |  | (0.0341) | (0.0407) |  | (0.0348) | (0.0409) | (0.0349) | (0.0412) | (0.0633) | (0.0459) |
| Gini disposable T-5 | -2.453 | -0.122 | -1.708** |  |  |  |  |  |  |  |
|  | (1.591) | (0.649) | (0.869) |  |  |  |  |  |  |  |
| Δ Redistribution T-5 |  |  |  | -239.5 | -111.2 | -306.6** |  |  |  |  |
|  |  |  |  | (146.1) | (116.9) | (141.3) |  |  |  |  |
| Δ Gini market T-5 |  |  |  |  |  |  | -6.423*** | -5.471* |  |  |
|  |  |  |  |  |  |  | (2.185) | (3.051) |  |  |
| Poverty T-5 |  |  |  |  |  |  |  |  | 102.1 | 177.6* |
|  |  |  |  |  |  |  |  |  | (103.7) | (104.3) |
| Δ GDP/cap. T-5 | 0.00422* | -9.52e-05 | 0.000830 | 0.00399 | -0.000125 | 0.000539 | -0.000341 | 0.000396 | 0.00152 | 0.00136 |
|  | (0.00250) | (0.00169) | (0.00245) | (0.00254) | (0.00169) | (0.00247) | (0.00168) | (0.00248) | (0.00126) | (0.00130) |
| Δ alcool T-5 | -0.0811 | 1.278 | 0.826 | -0.309 | 1.278 | 0.485 | 1.069 | 0.350 | 2.724 | -0.566 |
|  | (2.190) | (1.800) | (2.538) | (2.212) | (1.808) | (2.567) | (1.818) | (2.594) | (3.409) | (3.641) |
| Unemployment rate T-5 | 1.984* | 0.0908 | 0.767 | 1.805* | 0.140 | 0.678 | 0.468 | 0.976 | 0.917** | 0.969* |
|  | (1.092) | (0.454) | (0.617) | (1.079) | (0.451) | (0.608) | (0.454) | (0.642) | (0.447) | (0.499) |
| Δ pop. 65+ | -13.74 | -4.610 | 1.018 | -13.30 | -4.136 | 2.274 | -5.593 | 0.975 | 2.672 | 1.848 |
|  | (10.93) | (7.577) | (9.419) | (10.90) | (7.517) | (9.398) | (7.581) | (9.465) | (8.626) | (10.06) |
| Constant | 54,232*** | 9,511*** | 20,186*** | 54,492*** | 9,650*** | 20,920*** | 9,951*** | 21,081*** | 5,764*** | 12,162*** |
|  | (1,588) | (1,133) | (2,327) | (1,519) | (1,109) | (2,351) | (1,116) | (2,380) | (1,342) | (2,114) |
|  |  |  |  |  |  |  |  |  |  |  |
| Observations | 749 | 745 | 745 | 741 | 737 | 737 | 737 | 737 | 446 | 446 |
| R-squared | 0.972 | 0.983 | 0.988 | 0.972 | 0.983 | 0.988 | 0.983 | 0.988 | 0.982 | 0.987 |
| Number of ctyid | 20 | 20 | 20 | 20 | 20 | 20 | 20 | 20 | 20 | 20 |
| Standard errors in parentheses | |  |  |  |  |  |  |  |  |  |
| *** p<0.01, ** p<0.05, * p<0.1 | |  |  |  |  |  |  |  |  |  |
