# Supplementary material for: Welfare state decommodification and population health
Source: PLoS One. 2022 Aug 31;17(8):e0272698. doi: 10.1371/journal.pone.0272698 (PMC9432727; doi:10.1371/journal.pone.0272698)
Supplement: S1 File — (ZIP) [file pone.0272698.s001.zip › Table A11. Models adding lags at T-1 with Generosity and p90p10 .docx]

## Table A11. Models adding lags at T-1 with Generosity and p90p10

|  |  |  |  |  |  |  |  |  |
| --- | --- | --- | --- | --- | --- | --- | --- | --- |
|  | (1) | (2) | (3) | (4) | (5) | (6) | (7) | (8) |
|  | Women | | Men | | Women | | Men | |
|  |  |  |  |  |  |  |  |  |
| Lagged dependent variable | 0.606*** |  | 0.626*** |  | 0.696*** |  | 0.530*** |  |
|  | (0.0501) |  | (0.0361) |  | (0.0350) |  | (0.0425) |  |
| Generosity T-1 | 0.439 | 0.821 | 0.583 | 2.032 |  |  |  |  |
|  | (0.523) | (0.769) | (0.789) | (1.343) |  |  |  |  |
| Generosity T-5 | -2.096*** | -4.621*** | -1.786*** | -3.683*** |  |  |  |  |
|  | (0.531) | (0.633) | (0.614) | (0.932) |  |  |  |  |
| P90p10 T-1 |  |  |  |  | 2.622 | -14.33 | -8.511 | -19.35 |
|  |  |  |  |  | (7.662) | (10.36) | (11.09) | (15.33) |
| P90p10 T-5 |  |  |  |  | 18.68*** | 32.32*** | 43.40*** | 63.56*** |
|  |  |  |  |  | (5.627) | (7.754) | (9.724) | (13.18) |
| Δ GDP/cap. T-1 | 0.00164 | 0.00139 | 0.00191 | 0.00237 | 0.00177 | 0.00172 | 0.000949 | 0.000678 |
|  | (0.00150) | (0.00163) | (0.00144) | (0.00194) | (0.00114) | (0.00137) | (0.00151) | (0.00176) |
| Δ GDP/cap. T-5 | -0.00177 | -0.00162 | -0.00225 | -0.000966 | -0.00108 | -0.00138 | -0.00167 | -0.00106 |
|  | (0.00183) | (0.00199) | (0.00194) | (0.00256) | (0.00120) | (0.00141) | (0.00154) | (0.00178) |
| Δ alcohol T-1 | 2.185 | 2.129 | 3.641 | 2.364 | 2.591 | 1.875 | 2.042 | 1.259 |
|  | (2.151) | (1.935) | (2.430) | (2.405) | (2.549) | (2.475) | (3.571) | (3.561) |
| Δ alcohol T-5 | -4.133* | -1.743 | -6.063** | -4.525* | -0.172 | 2.190 | -0.544 | 1.822 |
|  | (2.187) | (2.021) | (2.423) | (2.483) | (2.221) | (2.157) | (3.267) | (3.356) |
| Unemployment rate T-1 | -0.00912 | -0.839 | 0.609 | 0.774 | -0.433 | -1.726*** | -0.185 | -0.514 |
|  | (0.466) | (0.653) | (0.467) | (0.788) | (0.343) | (0.542) | (0.473) | (0.700) |
| Unemployment rate T-5 | -0.0877 | 0.0487 | 0.561 | 3.252*** | 0.166 | 0.567 | 0.458 | 1.301* |
|  | (0.460) | (0.647) | (0.494) | (0.773) | (0.343) | (0.524) | (0.454) | (0.687) |
| Δ pop. 65+ T-1 | -28.61*** | -40.21*** | -31.52*** | -55.83*** | 7.195 | 20.38** | 16.48** | 27.46*** |
|  | (8.127) | (11.51) | (7.760) | (12.88) | (5.886) | (8.549) | (7.808) | (10.14) |
| Δ pop. 65+ T-5 | 17.47* | 6.758 | 43.02*** | 41.94*** | -0.0989 | -10.68 | 4.380 | -2.951 |
|  | (10.43) | (13.05) | (10.28) | (13.91) | (6.678) | (9.408) | (10.39) | (12.90) |
| Constant | 10,743*** | 28,655*** | 19,765*** | 55,046*** | 8,013*** | 25,956*** | 23,933*** | 50,944*** |
|  | (1,555) | (875.9) | (2,196) | (1,638) | (976.5) | (594.6) | (2,280) | (937.3) |
|  |  |  |  |  |  |  |  |  |
| Observations | 624 | 628 | 624 | 628 | 412 | 414 | 412 | 414 |
| R-squared | 0.986 | 0.971 | 0.990 | 0.978 | 0.990 | 0.975 | 0.993 | 0.985 |
| Number of countries | 20 | 20 | 20 | 20 | 20 | 20 | 20 | 20 |
| Standard errors in parentheses | | |  |  |  |  |  |  |
