# Supplementary material for: Welfare state decommodification and population health
Source: PLoS One. 2022 Aug 31;17(8):e0272698. doi: 10.1371/journal.pone.0272698 (PMC9432727; doi:10.1371/journal.pone.0272698)
Supplement: S1 File — (ZIP) [file pone.0272698.s001.zip › Table A10. Main models with life expectancy at birth as a dependent variable .docx]

Table A10. Main models with life expectancy at birth **as a dependent variable**

|  |  |  |  |  |  |  |  |  |  |  |
| --- | --- | --- | --- | --- | --- | --- | --- | --- | --- | --- |
|  | (1) | (2) | (3) | (4) | (5) | (6) | (7) | (8) | (9) | (10) |
|  | Women | Men | Women | Men | Women | Men | Women | Men | Women | Men |
|  |  |  |  |  |  |  |  |  |  |  |
| Lagged dependent variable | 0.708*** | 0.739*** | 0.662*** | 0.704*** | 0.383*** | 0.547*** | 0.793*** | 0.798*** | 0.798*** | 0.801*** |
|  | (0.0451) | (0.0299) | (0.0620) | (0.0593) | (0.108) | (0.0822) | (0.0368) | (0.0334) | (0.0356) | (0.0329) |
| Generosity T-5 | 0.00999* | 0.00292 |  |  |  |  |  |  |  |  |
|  | (0.00590) | (0.00480) |  |  |  |  |  |  |  |  |
| P90p10 T-5 |  |  | -0.188** | -0.180** |  |  |  |  |  |  |
|  |  |  | (0.0850) | (0.0862) |  |  |  |  |  |  |
| Risk reduction T-5 |  |  |  |  | 0.675** | 0.680*** |  |  |  |  |
|  |  |  |  |  | (0.307) | (0.248) |  |  |  |  |
| Δ Gini disp T-5 |  |  |  |  |  |  | 0.0189 | 0.0272 |  |  |
|  |  |  |  |  |  |  | (0.0237) | (0.0222) |  |  |
| Redis. T-5 |  |  |  |  |  |  |  |  | -0.157 | 0.370 |
|  |  |  |  |  |  |  |  |  | (0.479) | (0.511) |
| Δ GDP/cap. T-5 | -2.22e-05 | -2.45e-05 | -1.13e-05 | -1.00e-05 | -5.02e-05* | -3.73e-05* | -3.24e-06 | -1.61e-05 | -3.61e-06 | -1.60e-05 |
|  | (2.26e-05) | (1.51e-05) | (2.18e-05) | (1.65e-05) | (2.96e-05) | (2.15e-05) | (1.89e-05) | (1.64e-05) | (1.89e-05) | (1.65e-05) |
| Δ alcool T-5 | 0.0215 | 0.0325 | -0.0279 | -0.0171 | 0.00172 | 0.0221 | -0.00687 | 0.0121 | -0.00540 | 0.0130 |
|  | (0.0279) | (0.0225) | (0.0381) | (0.0322) | (0.0380) | (0.0334) | (0.0229) | (0.0206) | (0.0225) | (0.0202) |
| Unemployment rate T-5 | -0.00951 | -0.0161*** | -0.0123** | -0.0100** | -0.0446*** | -0.0308*** | -0.00711 | -0.0118*** | -0.00671 | -0.0113*** |
|  | (0.00729) | (0.00519) | (0.00564) | (0.00464) | (0.0101) | (0.00824) | (0.00509) | (0.00454) | (0.00497) | (0.00437) |
| Δ pop. 65+ | -0.0705 | -0.130 | -0.0895 | -0.177* | 0.127 | 0.0533 | -0.0499 | -0.0818 | -0.0504 | -0.0776 |
|  | (0.136) | (0.105) | (0.103) | (0.0947) | (0.113) | (0.112) | (0.0887) | (0.0797) | (0.0887) | (0.0792) |
| Constant | -89.20*** | -124.7*** | -102.6*** | -139.3*** | -43.20** | -80.00*** | -62.51*** | -96.22*** | -61.23*** | -95.13*** |
|  | (15.86) | (15.73) | (20.06) | (28.95) | (18.28) | (19.27) | (13.01) | (17.09) | (12.74) | (16.86) |
|  |  |  |  |  |  |  |  |  |  |  |
| Observations | 714 | 714 | 440 | 440 | 327 | 327 | 766 | 766 | 773 | 773 |
| R-squared | 0.991 | 0.995 | 0.997 | 0.997 | 0.990 | 0.995 | 0.997 | 0.997 | 0.997 | 0.997 |
| Number of countries | 20 | 20 | 20 | 20 | 18 | 18 | 20 | 20 | 20 | 20 |
| Standard errors in parentheses | | |  |  |  |  |  |  |  |  |
| *** p<0.01, ** p<0.05, * p<0.1 | | |  |  |  |  |  |  |  |  |
