# Supplementary material for: Welfare state decommodification and population health
Source: PLoS One. 2022 Aug 31;17(8):e0272698. doi: 10.1371/journal.pone.0272698 (PMC9432727; doi:10.1371/journal.pone.0272698)
Supplement: S1 File — (ZIP) [file pone.0272698.s001.zip › Table A9. Main models with life expectancy at 65 years old as a dependent variable .docx]

Table A9. Main models with life expectancy at 65 years old **as a dependent variable**

|  |  |  |  |  |  |  |  |  |  |  |
| --- | --- | --- | --- | --- | --- | --- | --- | --- | --- | --- |
|  | (1) | (2) | (3) | (4) | (5) | (6) | (7) | (8) | (9) | (10) |
|  | Women | Men | Women | Men | Women | Men | Women | Men | Women | Men |
|  |  |  |  |  |  |  |  |  |  |  |
| Lagged dependent variable | 0.630*** | 0.727*** | 0.609*** | 0.552*** | 0.397*** | 0.501*** | 0.751*** | 0.817*** | 0.757*** | 0.824*** |
|  | (0.0588) | (0.0415) | (0.0692) | (0.0608) | (0.123) | (0.105) | (0.0401) | (0.0371) | (0.0389) | (0.0363) |
| Generosity T-5 | 0.0131** | -0.00123 |  |  |  |  |  |  |  |  |
|  | (0.00598) | (0.00466) |  |  |  |  |  |  |  |  |
| P90p10 T-5 |  |  | -0.223*** | -0.257*** |  |  |  |  |  |  |
|  |  |  | (0.0808) | (0.0664) |  |  |  |  |  |  |
| Risk reduction T-5 |  |  |  |  | 0.541* | 0.522** |  |  |  |  |
|  |  |  |  |  | (0.315) | (0.215) |  |  |  |  |
| Δ Gini disp T-5 |  |  |  |  |  |  | 0.0225 | -0.00870 |  |  |
|  |  |  |  |  |  |  | (0.0225) | (0.0164) |  |  |
| Redis. T-5 |  |  |  |  |  |  |  |  | -0.443 | -0.237 |
|  |  |  |  |  |  |  |  |  | (0.411) | (0.312) |
| Δ GDP/cap. T-5 | -2.53e-05 | -2.28e-05 | -1.25e-05 | -1.10e-05 | -4.21e-05 | -3.60e-05* | -8.21e-06 | -8.23e-06 | -8.71e-06 | -8.10e-06 |
|  | (2.21e-05) | (1.54e-05) | (2.20e-05) | (1.55e-05) | (3.09e-05) | (2.15e-05) | (1.90e-05) | (1.44e-05) | (1.87e-05) | (1.42e-05) |
| Δ alcool T-5 | 0.0180 | 0.0271 | -0.0300 | -0.00666 | -0.00172 | 0.0215 | 0.00233 | 0.00679 | 0.00220 | 0.00764 |
|  | (0.0267) | (0.0207) | (0.0351) | (0.0250) | (0.0371) | (0.0295) | (0.0212) | (0.0162) | (0.0208) | (0.0159) |
| Unemployment rate T-5 | -0.0125* | -0.0160*** | -0.0117** | -0.0159*** | -0.0374*** | -0.0274*** | -0.00891* | -0.00832** | -0.00797* | -0.00826** |
|  | (0.00696) | (0.00515) | (0.00576) | (0.00414) | (0.0102) | (0.00767) | (0.00490) | (0.00359) | (0.00478) | (0.00347) |
| Δ pop. 65+ | -0.0193 | -0.0593 | -0.0441 | -0.0224 | 0.117 | 0.0889 | -0.0238 | -0.0551 | -0.0287 | -0.0536 |
|  | (0.131) | (0.0930) | (0.0969) | (0.0735) | (0.105) | (0.0908) | (0.0847) | (0.0617) | (0.0850) | (0.0612) |
| Constant | -93.10*** | -92.67*** | -102.8*** | -157.2*** | -61.13*** | -97.13*** | -60.79*** | -60.79*** | -59.42*** | -58.90*** |
|  | (15.37) | (14.06) | (18.23) | (20.60) | (22.04) | (26.98) | (10.45) | (12.24) | (10.18) | (11.89) |
|  |  |  |  |  |  |  |  |  |  |  |
| Observations | 711 | 711 | 440 | 440 | 327 | 327 | 762 | 762 | 769 | 769 |
| R-squared | 0.985 | 0.991 | 0.989 | 0.994 | 0.977 | 0.989 | 0.991 | 0.996 | 0.991 | 0.996 |
| Number of countries | 20 | 20 | 20 | 20 | 18 | 18 | 20 | 20 | 20 | 20 |
| Standard errors in parentheses | | |  |  |  |  |  |  |  |  |
| *** p<0.01, ** p<0.05, * p<0.1 | | |  |  |  |  |  |  |  |  |
