# Supplementary material for: Welfare state decommodification and population health
Source: PLoS One. 2022 Aug 31;17(8):e0272698. doi: 10.1371/journal.pone.0272698 (PMC9432727; doi:10.1371/journal.pone.0272698)
Supplement: S1 File — (ZIP) [file pone.0272698.s001.zip › Table A8. Main models without controls.docx]

## Table A8. Main models without controls

|  | (1) | (2) | (3) | (4) | (5) | (6) | (7) | (8) | (9) | (10) |
| --- | --- | --- | --- | --- | --- | --- | --- | --- | --- | --- |
|  | Women | Men | Women | Men | Women | Men | Women | Men | Women | Men |
|  |  |  |  |  |  |  |  |  |  |  |
| Lagged dependent variable | 0.636*** | 0.615*** | 0.553*** | 0.406*** | 0.766*** | 0.698*** | 0.753*** | 0.701*** | 0.532*** | 0.645*** |
|  | (0.0396) | (0.0324) | (0.0451) | (0.0515) | (0.0229) | (0.0262) | (0.0241) | (0.0275) | (0.0951) | (0.114) |
| Generosity T-5 | -1.657*** | -1.311*** |  |  |  |  |  |  |  |  |
|  | (0.460) | (0.479) |  |  |  |  |  |  |  |  |
| P90p10 T-5 |  |  | 22.12*** | 45.98*** |  |  |  |  |  |  |
|  |  |  | (6.784) | (10.77) |  |  |  |  |  |  |
| Redistribution T-5 |  |  |  |  | -46.18 | 29.66 |  |  |  |  |
|  |  |  |  |  | (34.34) | (47.65) |  |  |  |  |
| Δ Gini disp T-5 |  |  |  |  |  |  | -1.285 | -0.746 |  |  |
|  |  |  |  |  |  |  | (2.045) | (2.794) |  |  |
| Risk reduction T-5 |  |  |  |  |  |  |  |  | -9.891 | -60.93* |
|  |  |  |  |  |  |  |  |  | (23.66) | (34.07) |
| Constant | 9,222*** | 19,266*** | 11,613*** | 30,532*** | 6,030*** | 15,702*** | 6,392*** | 15,248*** | 4,778* | 5,125 |
|  | (1,165) | (1,821) | (1,284) | (2,721) | (786.0) | (1,603) | (808.1) | (1,651) | (2,702) | (4,406) |
|  |  |  |  |  |  |  |  |  |  |  |
| Observations | 731 | 731 | 432 | 432 | 842 | 842 | 822 | 822 | 312 | 312 |
| R-squared | 0.982 | 0.987 | 0.984 | 0.990 | 0.984 | 0.988 | 0.984 | 0.988 | 0.975 | 0.982 |
| Number of ctyid | 21 | 21 | 21 | 21 | 21 | 21 | 21 | 21 | 19 | 19 |
| Standard errors in parentheses *** p<0.01, ** p<0.05, * p<0.1 | | | | | | | | |  |  |
