# Supplementary material for: Welfare state decommodification and population health
Source: PLoS One. 2022 Aug 31;17(8):e0272698. doi: 10.1371/journal.pone.0272698 (PMC9432727; doi:10.1371/journal.pone.0272698)
Supplement: S1 File — (ZIP) [file pone.0272698.s001.zip › Table A7. Models replicating table 2 but restraining the samples based on risk reduction (models 1 to 4) or p90p10 (5 to 8).docx]

Table A7 is used to confirm the insignificant association between redistribution or disposable Gini and population health in samples that use only country-years for which we have the risk reduction measure (models 1 to 4) or the p90p10 measure (models 5 to 8). This is to confirm that the significant association of the p90p10 and the risk reduction measure is not determined by a smaller sample size.

Table A7. Models replicating table 2 but restraining the samples **based on risk reduction (models 1 to 4) or p90p10 (5 to 8)**

|  |  |  |  |  |  |  |  |  |
| --- | --- | --- | --- | --- | --- | --- | --- | --- |
|  | (1) | (2) | (3) | (4) | (5) | (6) | (7) | (8) |
|  | Women | Men | Women | Men | Women | Men | Women | Men |
|  |  |  |  |  |  |  |  |  |
| Lagged dependent variable | 0.460*** | 0.607*** | 0.461*** | 0.604*** | 0.566*** | 0.444*** | 0.572*** | 0.446*** |
|  | (0.0850) | (0.106) | (0.0868) | (0.106) | (0.0452) | (0.0490) | (0.0435) | (0.0490) |
| Δ Gini disp T-5 | -6.289 | -5.636 |  |  | -5.829** | -2.566 |  |  |
|  | (3.984) | (5.547) |  |  | (2.715) | (2.981) |  |  |
| Redis. T-5 |  |  | -101.9 | -108.7 |  |  | 42.46 | -27.84 |
|  |  |  | (196.6) | (293.8) |  |  | (86.34) | (106.8) |
| Δ GDP/cap. T-5 | 0.00199 | 0.00342 | 0.00220 | 0.00361 | -0.000715 | -0.000872 | -0.000714 | -0.000875 |
|  | (0.00215) | (0.00300) | (0.00218) | (0.00302) | (0.00132) | (0.00157) | (0.00131) | (0.00157) |
| Δ alcool T-5 | 1.070 | -1.909 | 0.722 | -2.266 | 1.195 | -1.287 | 1.135 | -1.366 |
|  | (2.685) | (3.965) | (2.949) | (4.256) | (2.135) | (3.267) | (2.180) | (3.293) |
| Unemployment rate T-5 | 3.736*** | 3.949*** | 3.663*** | 3.911*** | 0.500 | 0.370 | 0.206 | 0.278 |
|  | (0.799) | (1.140) | (0.851) | (1.199) | (0.427) | (0.534) | (0.436) | (0.544) |
| Δ pop. 65+ | -18.99** | -12.85 | -18.73* | -12.67 | 3.032 | 14.12 | 3.033 | 14.20 |
|  | (9.546) | (13.92) | (9.858) | (14.32) | (7.550) | (10.33) | (7.510) | (10.32) |
| Constant | 4,351* | 5,610 | 5,000* | 6,372 | 10,544*** | 27,016*** | 10,350*** | 26,933*** |
|  | (2,302) | (4,184) | (2,791) | (4,586) | (1,240) | (2,560) | (1,197) | (2,556) |
|  |  |  |  |  |  |  |  |  |
| Observations | 301 | 301 | 301 | 301 | 416 | 416 | 416 | 416 |
| R-squared | 0.976 | 0.982 | 0.976 | 0.982 | 0.984 | 0.989 | 0.983 | 0.989 |
| Number of countries | 18 | 18 | 18 | 18 | 20 | 20 | 20 | 20 |
| Standard errors in parentheses | | |  |  |  |  |  |  |
| *** p<0.01, ** p<0.05, * p<0.1 | | |  |  |  |  |  |  |
