# Supplementary material for: Welfare state decommodification and population health
Source: PLoS One. 2022 Aug 31;17(8):e0272698. doi: 10.1371/journal.pone.0272698 (PMC9432727; doi:10.1371/journal.pone.0272698)
Supplement: S1 File — (ZIP) [file pone.0272698.s001.zip › Table A6. Effect of different measures of risk reduction.docx]

Table A6 uses alternative measures of risk reduction. Measures including the whole population are less clearly correlated with population health. This is not surprising, since Hacker and Rehm (2020) argue that their measure is specifically designed to capture shocks to income for the working age population because income dynamics after retirement are not appropriate measures of risk, given the important of wealth for post-retirement well-being. Moreover, shocks to income are much less frequent for retirees. The measure of risk reduction at 50% of income losses for the working age population is significantly related to population health, for men, but not for women, like for the measure of 25% of income losses.

| Table A6. Effect of different measures of risk reduction  \|  \| (1) \| (2) \| (3) \| (4) \| (5) \| (6) \| (7) \| (8) \| \| --- \| --- \| --- \| --- \| --- \| --- \| --- \| --- \| --- \| \|  \| Women \| Men \| Women \| Men \| Women \| Men \| Women \| Men \| \|  \|  \|  \|  \|  \|  \|  \|  \|  \| \| Lagged dependent variable \| 0.457*** \| 0.586*** \| 0.464*** \| 0.597*** \| 0.461*** \| 0.583*** \| 0.421*** \| 0.502*** \| \|  \| (0.0858) \| (0.107) \| (0.0857) \| (0.106) \| (0.0857) \| (0.105) \| (0.0908) \| (0.125) \| \| Risk reduction all pop. 25% T-5 \| -31.73 \| -66.32* \|  \|  \|  \|  \|  \|  \| \|  \| (20.25) \| (34.53) \|  \|  \|  \|  \|  \|  \| \| Risk reduction all pop. 50% T-5 \|  \|  \| -23.43 \| -59.69** \|  \|  \|  \|  \| \|  \|  \|  \| (17.48) \| (30.40) \|  \|  \|  \|  \| \| Risk reduction working age 50% T-5 \|  \|  \|  \|  \| -22.76 \| -71.19** \| -13.75 \| -76.11** \| \|  \|  \|  \|  \|  \| (17.67) \| (28.01) \| (20.25) \| (31.28) \| \| Generosity T-5 \|  \|  \|  \|  \|  \|  \| -4.844*** \| -6.665** \| \|  \|  \|  \|  \|  \|  \|  \| (1.665) \| (2.607) \| \| Δ GDP/cap. T-5 \| 0.00200 \| 0.00319 \| 0.00206 \| 0.00332 \| 0.00222 \| 0.00372 \| 0.00285 \| 0.00415 \| \|  \| (0.00212) \| (0.00296) \| (0.00214) \| (0.00295) \| (0.00214) \| (0.00292) \| (0.00217) \| (0.00294) \| \| Δ alcool T-5 \| 1.265 \| -1.414 \| 0.992 \| -1.970 \| 0.801 \| -2.573 \| -0.813 \| -5.338 \| \|  \| (2.670) \| (3.935) \| (2.704) \| (3.930) \| (2.761) \| (3.976) \| (2.953) \| (4.129) \| \| Unemployment rate T-5 \| 3.734*** \| 4.197*** \| 3.711*** \| 4.209*** \| 3.735*** \| 4.394*** \| 3.803*** \| 4.587*** \| \|  \| (0.834) \| (1.229) \| (0.825) \| (1.213) \| (0.825) \| (1.177) \| (0.907) \| (1.286) \| \| Δ pop. 65+ \| -19.93** \| -15.16 \| -19.43** \| -14.39 \| -19.75** \| -15.80 \| -19.81* \| -13.21 \| \|  \| (9.713) \| (13.98) \| (9.881) \| (14.29) \| (9.841) \| (14.15) \| (10.67) \| (14.95) \| \| Constant \| 4,128* \| 5,603 \| 4,228* \| 5,648 \| 4,160* \| 5,631 \| 4,356* \| 8,289* \| \|  \| (2,331) \| (4,035) \| (2,387) \| (4,112) \| (2,331) \| (3,944) \| (2,616) \| (4,931) \| \|  \|  \|  \|  \|  \|  \|  \|  \|  \| \| Observations \| 301 \| 301 \| 301 \| 301 \| 301 \| 301 \| 285 \| 285 \| \| R-squared \| 0.976 \| 0.982 \| 0.976 \| 0.982 \| 0.976 \| 0.982 \| 0.977 \| 0.983 \| \| Number of countries \| 18 \| 18 \| 18 \| 18 \| 18 \| 18 \| 18 \| 18 \| \| Standard errors in parentheses \| \| \|  \|  \|  \|  \|  \|  \| \| *** p<0.01, ** p<0.05, * p<0.1 \| \| \|  \|  \|  \|  \|  \|  \| |  |
| --- | --- | --- | --- | --- | --- | --- | --- | --- | --- | --- | --- | --- | --- | --- | --- | --- | --- | --- | --- | --- | --- | --- | --- | --- | --- | --- | --- | --- | --- | --- | --- | --- | --- | --- | --- | --- | --- | --- | --- | --- | --- | --- | --- | --- | --- | --- | --- | --- | --- | --- | --- | --- | --- | --- | --- | --- | --- | --- | --- | --- | --- | --- | --- | --- | --- | --- | --- | --- | --- | --- | --- | --- | --- | --- | --- | --- | --- | --- | --- | --- | --- | --- | --- | --- | --- | --- | --- | --- | --- | --- | --- | --- | --- | --- | --- | --- | --- | --- | --- | --- | --- | --- | --- | --- | --- | --- | --- | --- | --- | --- | --- | --- | --- | --- | --- | --- | --- | --- | --- | --- | --- | --- | --- | --- | --- | --- | --- | --- | --- | --- | --- | --- | --- | --- | --- | --- | --- | --- | --- | --- | --- | --- | --- | --- | --- | --- | --- | --- | --- | --- | --- | --- | --- | --- | --- | --- | --- | --- | --- | --- | --- | --- | --- | --- | --- | --- | --- | --- | --- | --- | --- | --- | --- | --- | --- | --- | --- | --- | --- | --- | --- | --- | --- | --- | --- | --- | --- | --- | --- | --- | --- | --- | --- | --- | --- | --- | --- | --- | --- | --- | --- | --- | --- | --- | --- | --- | --- | --- | --- | --- | --- | --- | --- | --- | --- | --- | --- | --- | --- | --- | --- | --- | --- | --- | --- | --- | --- | --- | --- | --- | --- | --- | --- | --- | --- | --- | --- | --- | --- | --- | --- | --- | --- | --- | --- | --- | --- | --- | --- | --- | --- | --- | --- | --- | --- | --- | --- | --- | --- | --- | --- | --- |
