# Supplementary material for: Welfare state decommodification and population health
Source: PLoS One. 2022 Aug 31;17(8):e0272698. doi: 10.1371/journal.pone.0272698 (PMC9432727; doi:10.1371/journal.pone.0272698)
Supplement: S1 File — (ZIP) [file pone.0272698.s001.zip › Table A5. Models replicating Table 1 with the share of private health care expenditures and with public health care expenditures.docx]

| Table A5. Models replicating Table 1 with the share of private health care expenditures and with public health care expenditures  \|  \|  \|  \|  \|  \|  \|  \|  \|  \| \| --- \| --- \| --- \| --- \| --- \| --- \| --- \| --- \| --- \| \|  \| (1) \| (2) \| (3) \| (4) \| (5) \| (6) \| (7) \| (8) \| \|  \| Women \| Men \| Women \| Men \| Women \| Men \| Women \| Men \| \|  \|  \|  \|  \|  \|  \|  \|  \|  \| \| Lagged dependent variable \| 0.620*** \| 0.605*** \| 0.614*** \| 0.602*** \| 0.598*** \| 0.600*** \| 0.609*** \| 0.600*** \| \|  \| (0.0389) \| (0.0330) \| (0.0370) \| (0.0344) \| (0.0380) \| (0.0324) \| (0.0368) \| (0.0343) \| \| Generosity T-5 \| -1.578*** \| -1.381*** \|  \|  \|  \|  \|  \|  \| \|  \| (0.448) \| (0.477) \|  \|  \|  \|  \|  \|  \| \| Unemployment gen. T-5 \|  \|  \| -0.963 \| -0.712 \|  \|  \|  \|  \| \|  \|  \|  \| (1.048) \| (1.102) \|  \|  \|  \|  \| \| Pensions gen T-5 \|  \|  \|  \|  \| -5.278*** \| -4.038*** \|  \|  \| \|  \|  \|  \|  \|  \| (0.935) \| (0.933) \|  \|  \| \| Sickness gen. T-5 \|  \|  \|  \|  \|  \|  \| -1.241 \| -0.543 \| \|  \|  \|  \|  \|  \|  \|  \| (0.964) \| (1.252) \| \| Δ Public healthcare exp. T-5 \| -4.218 \| -4.947 \| 0.453 \| -0.210 \| -5.479 \| -5.162 \| 0.782 \| -0.0185 \| \|  \| (3.657) \| (4.304) \| (3.407) \| (4.222) \| (3.664) \| (4.273) \| (3.381) \| (4.199) \| \| Δ Share private healthcare exp. T-5 \| 0.301 \| 0.764 \| 0.544 \| 0.877 \| 0.148 \| 0.666 \| 0.546 \| 0.834 \| \|  \| (0.418) \| (0.513) \| (0.403) \| (0.540) \| (0.415) \| (0.508) \| (0.401) \| (0.540) \| \| Δ GDP/cap. T-5 \| -0.00113 \| -0.000802 \| -0.000458 \| -0.000129 \| -0.00184 \| -0.00117 \| -0.000350 \| -2.53e-05 \| \|  \| (0.00135) \| (0.00159) \| (0.00142) \| (0.00176) \| (0.00134) \| (0.00157) \| (0.00142) \| (0.00176) \| \| Δ alcool T-5 \| -1.592 \| -4.750* \| -0.389 \| -2.642 \| -0.179 \| -3.172 \| -0.567 \| -2.773 \| \|  \| (2.069) \| (2.696) \| (1.740) \| (2.303) \| (1.974) \| (2.605) \| (1.709) \| (2.289) \| \| Unemployment rate T-5 \| 0.408 \| 0.963** \| 0.368 \| 1.030** \| 0.668* \| 1.312*** \| 0.316 \| 1.038** \| \|  \| (0.359) \| (0.422) \| (0.418) \| (0.493) \| (0.351) \| (0.411) \| (0.420) \| (0.484) \| \| Δ pop. 65+ \| -0.167 \| 12.78 \| -1.385 \| 6.105 \| 0.0591 \| 12.13 \| -0.00827 \| 6.551 \| \|  \| (7.611) \| (8.045) \| (7.320) \| (8.032) \| (7.558) \| (8.009) \| (7.347) \| (7.782) \| \| Constant \| 9,219*** \| 19,040*** \| 9,374*** \| 18,988*** \| 9,660*** \| 19,208*** \| 9,502*** \| 19,083*** \| \|  \| (1,137) \| (1,845) \| (1,093) \| (1,895) \| (1,110) \| (1,813) \| (1,086) \| (1,893) \| \|  \|  \|  \|  \|  \|  \|  \|  \|  \| \| Observations \| 659 \| 659 \| 700 \| 700 \| 665 \| 665 \| 699 \| 699 \| \| R-squared \| 0.982 \| 0.987 \| 0.984 \| 0.988 \| 0.982 \| 0.987 \| 0.984 \| 0.988 \| \| Number of ctyid \| 20 \| 20 \| 20 \| 20 \| 20 \| 20 \| 20 \| 20 \| \| Standard errors in parentheses \| \|  \|  \|  \|  \|  \|  \|  \| \| *** p<0.01, ** p<0.05, * p<0.1 \| \|  \|  \|  \|  \|  \|  \|  \| |
| --- | --- | --- | --- | --- | --- | --- | --- | --- | --- | --- | --- | --- | --- | --- | --- | --- | --- | --- | --- | --- | --- | --- | --- | --- | --- | --- | --- | --- | --- | --- | --- | --- | --- | --- | --- | --- | --- | --- | --- | --- | --- | --- | --- | --- | --- | --- | --- | --- | --- | --- | --- | --- | --- | --- | --- | --- | --- | --- | --- | --- | --- | --- | --- | --- | --- | --- | --- | --- | --- | --- | --- | --- | --- | --- | --- | --- | --- | --- | --- | --- | --- | --- | --- | --- | --- | --- | --- | --- | --- | --- | --- | --- | --- | --- | --- | --- | --- | --- | --- | --- | --- | --- | --- | --- | --- | --- | --- | --- | --- | --- | --- | --- | --- | --- | --- | --- | --- | --- | --- | --- | --- | --- | --- | --- | --- | --- | --- | --- | --- | --- | --- | --- | --- | --- | --- | --- | --- | --- | --- | --- | --- | --- | --- | --- | --- | --- | --- | --- | --- | --- | --- | --- | --- | --- | --- | --- | --- | --- | --- | --- | --- | --- | --- | --- | --- | --- | --- | --- | --- | --- | --- | --- | --- | --- | --- | --- | --- | --- | --- | --- | --- | --- | --- | --- | --- | --- | --- | --- | --- | --- | --- | --- | --- | --- | --- | --- | --- | --- | --- | --- | --- | --- | --- | --- | --- | --- | --- | --- | --- | --- | --- | --- | --- | --- | --- | --- | --- | --- | --- | --- | --- | --- | --- | --- | --- | --- | --- | --- | --- | --- | --- | --- | --- | --- | --- | --- | --- | --- | --- | --- | --- | --- | --- | --- | --- | --- | --- | --- | --- | --- | --- | --- | --- | --- | --- | --- | --- | --- | --- | --- | --- | --- | --- | --- | --- | --- | --- | --- | --- | --- | --- | --- | --- | --- | --- | --- | --- | --- | --- | --- | --- | --- | --- | --- | --- | --- | --- | --- | --- | --- | --- | --- | --- | --- | --- | --- | --- | --- | --- | --- | --- | --- | --- | --- | --- | --- |
