# Supplementary material for: Welfare state decommodification and population health
Source: PLoS One. 2022 Aug 31;17(8):e0272698. doi: 10.1371/journal.pone.0272698 (PMC9432727; doi:10.1371/journal.pone.0272698)
Supplement: S1 File — (ZIP) [file pone.0272698.s001.zip › Table A4. Replication of Table 3, without lagged dependent variables .docx]

Table A4. Replication of Table 3, without lagged dependent variables

|  |  |  |  |  |  |  |  |  |
| --- | --- | --- | --- | --- | --- | --- | --- | --- |
|  | (1) | (2) | (3) | (4) | (5) | (6) | (7) | (8) |
|  | Women | Men | Women | Men | Women | Men | Women | Men |
|  |  |  |  |  |  |  |  |  |
| Generosity t-5 | -5.019*** | -4.642*** | -5.970*** | -4.519*** | -2.809*** | -1.692 | -6.381*** | -9.228*** |
|  | (0.656) | (0.738) | (0.750) | (0.891) | (0.687) | (1.042) | (1.967) | (2.888) |
| Δ Gini disp T-1 | -3.315 | -1.666 |  |  |  |  |  |  |
|  | (2.322) | (2.674) |  |  |  |  |  |  |
| Δ Gini disp T-5 | -4.825** | -0.177 |  |  |  |  |  |  |
|  | (2.239) | (2.632) |  |  |  |  |  |  |
| Redis T-1 |  |  | 199.6** | -161.0 |  |  |  |  |
|  |  |  | (97.63) | (119.2) |  |  |  |  |
| Redis. T-5 |  |  | 115.8 | 35.97 |  |  |  |  |
|  |  |  | (84.54) | (106.4) |  |  |  |  |
| p90p10 t-1 |  |  |  |  | -10.12 | -10.20 |  |  |
|  |  |  |  |  | (11.91) | (15.83) |  |  |
| P90p10 T-5 |  |  |  |  | 28.04*** | 56.94*** |  |  |
|  |  |  |  |  | (9.168) | (14.35) |  |  |
| Risk reduction T-1 |  |  |  |  |  |  | -9.488 | -30.86 |
|  |  |  |  |  |  |  | (21.20) | (32.77) |
| Risk reduction T-5 |  |  |  |  |  |  | -24.26 | -103.7*** |
|  |  |  |  |  |  |  | (23.60) | (37.02) |
| Δ GDP/cap. T-5 | 0.00178 | 0.00359** | 0.00182 | 0.00351** | -0.000913 | -0.00168 | 0.00457** | 0.00405 |
|  | (0.00145) | (0.00172) | (0.00146) | (0.00172) | (0.00150) | (0.00185) | (0.00224) | (0.00267) |
| Δ alcool T-5 | -0.496 | -3.306 | -0.314 | -3.305 | 2.177 | -0.0153 | -0.418 | -2.420 |
|  | (1.873) | (2.158) | (1.884) | (2.149) | (2.333) | (3.547) | (2.011) | (3.274) |
| Unemployment rate T-5 | 0.565 | 1.890** | 0.372 | 1.922** | 0.626 | 1.138 | 4.429*** | 5.883*** |
|  | (0.617) | (0.791) | (0.629) | (0.801) | (0.569) | (0.722) | (1.083) | (1.294) |
| Δ pop. 65+ | -9.257 | 2.205 | -7.584 | 2.537 | -9.189 | 5.838 | -23.04* | -24.41 |
|  | (9.780) | (10.78) | (9.827) | (10.85) | (9.988) | (13.46) | (12.90) | (15.83) |
| Constant | 27,113*** | 52,354*** | 27,032*** | 52,401*** | 25,888*** | 51,547*** | 10,264*** | 24,367*** |
|  | (809.5) | (1,389) | (843.1) | (1,383) | (741.4) | (1,149) | (2,907) | (4,289) |
|  |  |  |  |  |  |  |  |  |
| Observations | 669 | 669 | 675 | 675 | 401 | 401 | 281 | 281 |
| R-squared | 0.967 | 0.975 | 0.967 | 0.975 | 0.974 | 0.986 | 0.977 | 0.981 |
| Number of countries | 20 | 20 | 20 | 20 | 20 | 20 | 18 | 18 |
| Standard errors in parentheses | | |  |  |  |  |  |  |
| *** p<0.01, ** p<0.05, * p<0.1 | | |  |  |  |  |  |  |
