# Supplementary material for: Welfare state decommodification and population health
Source: PLoS One. 2022 Aug 31;17(8):e0272698. doi: 10.1371/journal.pone.0272698 (PMC9432727; doi:10.1371/journal.pone.0272698)
Supplement: S1 File — (ZIP) [file pone.0272698.s001.zip › Table A3. Replication of Table 2 without lagged dependent variables.docx]

| Table A3. Replication of Table 2 without lagged dependent variables | | | | | | | | |
| --- | --- | --- | --- | --- | --- | --- | --- | --- |
|  | (1) | (2) | (3) | (4) | (5) | (6) | (7) | (8) |
|  | Women | Men | Women | Men | Women | Men | Women | Men |
|  |  |  |  |  |  |  |  |  |
| P90p10 T-5 | 33.74*** | 60.70*** |  |  |  |  |  |  |
|  | (8.471) | (13.78) |  |  |  |  |  |  |
| Risk reduction T-5 |  |  | -43.52* | -114.6*** |  |  |  |  |
|  |  |  | (22.57) | (36.52) |  |  |  |  |
| Δ Gini disp T-5 |  |  |  |  | -4.374* | 0.241 |  |  |
|  |  |  |  |  | (2.367) | (3.289) |  |  |
| Redis. T-5 |  |  |  |  |  |  | -38.06 | -239.5 |
|  |  |  |  |  |  |  | (119.0) | (146.1) |
| Δ GDP/cap. T-5 | -0.000422 | -0.000309 | 0.00314 | 0.00414 | 0.00178 | 0.00396 | 0.00185 | 0.00399 |
|  | (0.00150) | (0.00189) | (0.00217) | (0.00312) | (0.00165) | (0.00253) | (0.00166) | (0.00254) |
| Δ alcool T-5 | 2.850 | 1.513 | 1.813 | 0.280 | 1.615 | -0.205 | 1.529 | -0.309 |
|  | (2.184) | (3.380) | (2.387) | (3.672) | (1.659) | (2.218) | (1.648) | (2.212) |
| Unemployment rate T-5 | 0.557 | 1.071 | 4.924*** | 7.065*** | 0.0169 | 1.802* | -0.0860 | 1.805* |
|  | (0.597) | (0.730) | (0.980) | (1.515) | (0.728) | (1.080) | (0.739) | (1.079) |
| Δ pop. 65+ | 0.0468 | 11.40 | -25.07** | -30.42* | -14.90* | -13.54 | -14.45* | -13.30 |
|  | (8.899) | (12.44) | (11.46) | (17.77) | (8.600) | (10.88) | (8.561) | (10.90) |
| Constant | 26,089*** | 51,293*** | 9,618*** | 20,639*** | 29,216*** | 54,471*** | 29,254*** | 54,492*** |
|  | (604.7) | (904.4) | (2,260) | (3,479) | (938.2) | (1,515) | (958.2) | (1,519) |
|  |  |  |  |  |  |  |  |  |
| Observations | 418 | 418 | 304 | 304 | 741 | 741 | 741 | 741 |
| R-squared | 0.974 | 0.984 | 0.974 | 0.980 | 0.964 | 0.972 | 0.963 | 0.972 |
| Number of ctyid | 20 | 20 | 18 | 18 | 20 | 20 | 20 | 20 |
| Standard errors in parentheses | |  |  |  |  |  |  |  |
| *** p<0.01, ** p<0.05, * p<0.1 | |  |  |  |  |  |  |  |
