# Supplementary material for: Welfare state decommodification and population health
Source: PLoS One. 2022 Aug 31;17(8):e0272698. doi: 10.1371/journal.pone.0272698 (PMC9432727; doi:10.1371/journal.pone.0272698)
Supplement: S1 File — (ZIP) [file pone.0272698.s001.zip › Table A2. Replication of Table 1 without lagged dependent variables.docx]

Table A2. Replication of Table 1 without lagged dependent variables

|  |  |  |  |  |  |  |  |  |
| --- | --- | --- | --- | --- | --- | --- | --- | --- |
|  | (1) | (2) | (3) | (4) | (5) | (6) | (7) | (8) |
|  | Women | Men | Women | Men | Women | Men | Women | Men |
|  |  |  |  |  |  |  |  |  |
| Lagged dependent variable | -4.349*** | -2.329*** |  |  |  |  |  |  |
|  | (0.521) | (0.699) |  |  |  |  |  |  |
| Generosity T-5 |  |  | -1.677 | 1.402 |  |  |  |  |
|  |  |  | (1.414) | (1.990) |  |  |  |  |
| Unemployment gen. T-5 |  |  |  |  | -14.80*** | -8.978*** |  |  |
|  |  |  |  |  | (1.294) | (1.643) |  |  |
| Pensions gen T-5 |  |  |  |  |  |  | -5.487*** | -2.980 |
|  |  |  |  |  |  |  | (1.435) | (2.217) |
| Sickness gen. T-5 | -0.000203 | 0.00149 | -3.71e-05 | 0.00172 | -0.000968 | 0.00119 | 0.000160 | 0.00196 |
|  | (0.00193) | (0.00253) | (0.00184) | (0.00304) | (0.00197) | (0.00256) | (0.00183) | (0.00306) |
| Δ GDP/cap. T-5 | 0.139 | -4.495 | 2.424 | -0.365 | 1.254 | -3.369 | 1.314 | -1.074 |
|  | (2.933) | (3.457) | (2.188) | (3.078) | (2.665) | (3.303) | (2.154) | (3.078) |
| Δ alcool T-5 | 0.254 | 2.871*** | -0.301 | 2.701*** | 1.148** | 3.810*** | -0.550 | 2.603*** |
|  | (0.510) | (0.678) | (0.548) | (0.827) | (0.482) | (0.633) | (0.542) | (0.815) |
| Unemployment rate T-5 | -12.73 | 5.924 | -5.156 | 7.545 | -4.086 | 10.42 | -2.514 | 4.226 |
|  | (10.80) | (12.85) | (11.38) | (13.61) | (10.36) | (12.48) | (11.14) | (13.87) |
| Δ pop. 65+ | 26,905*** | 52,148*** | 26,628*** | 51,441*** | 26,497*** | 51,918*** | 26,627*** | 51,450*** |
|  | (608.9) | (1,174) | (576.6) | (1,098) | (620.3) | (1,195) | (554.8) | (1,076) |
|  |  |  |  |  |  |  |  |  |
| Observations | 707 | 707 | 753 | 753 | 715 | 715 | 752 | 752 |
| R-squared | 0.970 | 0.980 | 0.971 | 0.981 | 0.972 | 0.980 | 0.972 | 0.981 |
| Number of countries | 20 | 20 | 20 | 20 | 20 | 20 | 20 | 20 |
| Standard errors in parentheses | | |  |  |  |  |  |  |
| *** p<0.01, ** p<0.05, * p<0.1 | | |  |  |  |  |  |  |
